# Genome-wide CRISPR Screen Reveals RAB10 as a Synthetic Lethal Gene in Colorectal and Pancreatic Cancers Carrying SMAD4 Loss

**DOI:** 10.1158/2767-9764.CRC-22-0301

**Published:** 2023-05-04

**Authors:** Hélène Erasimus, Vanessa Kolnik, Frédéric Lacroix, Sukhvinder Sidhu, Stéphane D'Agostino, Olivier Lemaitre, Alexandre Rohaut, Isabelle Sanchez, Gilbert Thill, Michel Didier, Laurent Debussche, Christophe Marcireau

**Affiliations:** 1 Sanofi, Molecular Oncology, Vitry-sur-Seine, France.; 2 Sanofi, Oncology, *in vivo* Pharmacology, Vitry-Sur-Seine, France.; 3 Sanofi, Translational Sciences, Chilly-Mazarin, France.

## Abstract

The TGFβ signaling mediator SMAD4 is frequently mutated or deleted in colorectal and pancreatic cancers. SMAD4 acts as a tumor suppressor and its loss is associated with poorer patient outcomes. The purpose of this study was to find synthetic lethal interactions with SMAD4 deficiency to find novel therapeutic strategies for the treatment of patients with SMAD4-deficient colorectal or pancreatic cancers. Using pooled lentiviral single-guide RNA libraries, we conducted genome-wide loss-of-function screens in Cas9-expressing colorectal and pancreatic cancer cells harboring altered or wild-type SMAD4. The small GTPase protein RAB10 was identified and validated as a susceptibility gene in SMAD4-altered colorectal and pancreatic cancer cells. Rescue assays showed that RAB10 reintroduction reversed the antiproliferative effects of RAB10 knockout in SMAD4-negative cell lines. Further investigation is necessary to shed light on the mechanism by which RAB10 inhibition decreases cell proliferation of SMAD4-negative cells.

**Significance:**

This study identified and validated RAB10 as new synthetic lethal gene with SMAD4. This was achieved by conducting a whole-genome CRISPR screens in different colorectal and pancreatic cell lines. A future RAB10 inhibitors could correspond to a new therapeutic solution for patients with cancer with SMAD4 deletion.

## Introduction

TGFβ pathway is an important signaling process that regulates the homeostasis of epithelial, stromal, or immune cells (1). It integrates signaling from the cell membrane to the nucleus. Upon TGFβ receptor activation, SMAD2 and SMAD3 proteins are phosphorylated and form a complex with SMAD4. This complex is then translocated into the nucleus where it binds SMAD binding elements of target genes. With other transcription coactivators, SMAD2/3/4 complex mediates the TGFβ-induced transcriptional program. Disregulation of this pathway contributes to different pathologies such as cancer, fibrosis, nonalcoholic steatohepatitis, or inflammatory diseases. A pan-cancer genomic analysis revealed that genetic alterations of the TGFβ pathway are observed at high frequency in cancers [39% of The Cancer Genome Atlas (TCGA) cases] (2).

Among the key players of the TGFβ signaling pathway, the well-known tumor suppressor gene SMAD4 is often lost via mutations/deletions. Juvenile polyposis syndrome (JPS) is a hereditary gastrointestinal polyposis syndrome where SMAD4 germline mutation is found in 20% of cases (3). Patients with JPS have a higher risk of developing cancers of the gastrointestinal tract (4, 5). SMAD4 genetic alterations, either via genomic loss or via mutations, can be found in many cancer types (6). For example, SMAD4 is frequently mutated in esophageal carcinoma (7), or ampullary carcinoma (8). In squamous cell carcinomas, SMAD4 genetic loss is often observed, rather than mutation (9). SMAD4 is also mutated or deleted in 10%–15% and 20%–50% of colorectal and pancreatic cancers, respectively (10–12), in which its loss of expression is associated with inferior outcomes in patients with colorectal cancer (13–15) and pancreatic cancer (16–18). Moreover, SMAD4 inactivation correlates with the presence of metastatic disease in colorectal cancer (19) as well as pancreatic cancer (20). In addition to its prognostic value, loss of SMAD4 expression has been shown to be associated with worse response to fluorouracil-based treatment in colorectal cancer (13) and to concurrent chemotherapy along with local control treatment in a subset of patients with pancreatic cancer with locoregional recurrences (18). This makes the context of SMAD4 loss an attractive target to identify synthetic lethal interactions and new therapeutic options.

In cancers, TGFβ can have opposite roles depending on cellular context. It can act as a tumor promoter or tumor suppressor. Canonical TGFβ/SMAD4 signaling behaves like a tumor suppressor pathway in early stages of tumorigenesis in most tissues. Cancer cell proliferation is inhibited by this TGFβ signaling, which requires functional SMAD4. In this context, TGFβ/SMAD4 activation induces cell-cycle G_1_–S arrest (21) and apoptosis (22). In later cancer stages, tumor progression is stimulated by TGFβ. This effect can be mediated by change in the tumor microenvironment such as epithelial–mesenchymal transition, changes in the extracellular matrix, angiogenesis, and effects on the immune system (23). In the immune system, TGFβ controls immune tolerance and inflammation. TGFβ signaling is involved in tumor immune evasion through different mechanisms such as the inhibition of T cell–dependent cytotoxic responses, inhibition of TH1 cells, as well as the stimulation of immunosuppressive regulatory T cells. In addition, natural killer–cell antitumor activity can also be inhibited by TGFβ in the tumor microenvironment.

The development of therapies based on TGFβ pathway inhibition was boosted with the emergence of immunotherapy. Different small molecules, mAbs, or ligand traps have shown interesting results in preclinical studies and are currently being tested in clinical trials (1, 24). In general, these therapeutic agents act to decrease the tumor promoting aspects of the TGFβ pathway, without distinction between cancerous and healthy tissues.

To date, to the best of our knowledge, there is no therapeutic approach taking advantage of the genetic context of the high frequency of mutations at the SMAD4 locus. Loss of SMAD4 function/protein could be harnessed by identifying synthetic lethal genes that encode for druggable proteins. Genes are synthetic lethal partners, when their combined perturbation results in cell death, while their individual perturbation is well tolerated. The synthetic lethality approach has the opportunity to make SMAD4 an actionable target by targeting specifically SMAD4-negative tumor cells, while limiting harm to normal cells. Synthetic lethality has become a valuable concept, as shown by successful advances in the treatment of BRCA1/BRCA2-negative ovarian/breast/prostate cancers with the use of PARP inhibitors. Other examples of synthetic lethal drugs currently evaluated in clinical trials include ATR, CHK1, and WEE1 inhibitors (25).

Some studies have nevertheless paved the way for taking advantage of SMAD4 loss. As an example, the Kruppel like factor 5 (KLF5), is a potentially interesting therapeutic target in SMAD4-negative colorectal and pancreatic cancers. KLF5 has been shown to promote cancer cell proliferation in various cancers (26, 27). High expression of KLF5 is associated with poor survival in colorectal and pancreatic cancers (28, 29). It has been shown that KLF5 inhibition can restore oxaliplatin sensitivity in patient-derived colorectal cancer organoids by restoring the apoptotic response (30). One study showed that KLF5 inhibition in SMAD4-mutant pancreatic ductal adenocarcinoma cells is accompanied by increased apoptosis (31). Another study showed that KLF5 knockdown in SMAD4-deficient colorectal cancer cells increases TGFβ-induced apoptosis and makes the cells more sensitive to 5-fluorouracil treatment (32). Similarly, Shen and colleagues showed that knockdown of KLF5 in SW620 cells lacking SMAD4 promoted cell apoptosis (30). Other genes, such as Aurora kinase A (AURKA) and members of the bromodomain and extraterminal motif (BET), BRD2 and BRD4, have also recently been described as potentially synthetic lethal genes with SMAD4 loss (33, 34). An additional option previously described consists of taking advantage of a “collateral lethality” which is often observed in a SMAD4-negative setting. Loss of the metabolic gene malic enzyme 2 (ME2) in the SMAD4 locus has been shown to create a cancer-specific metabolic vulnerability upon targeting of its paralogous isoform ME3 in pancreatic ductal adenocarcinoma lacking SMAD4 (35).

CRISPR/CAS9 viability screen is a useful tool to pinpoint new synthetic lethality interactions (36). In this study, we identified and characterized four nonisogenic colorectal and pancreatic cancer cell lines harboring wild-type (DLD1 and MIAPaCa-2) and mutant (HT29 and SW620) SMAD4, and we identified SMAD4-dependent genetic vulnerabilities through an unbiased, genome-wide, loss-of-function CRISPR/Cas9 screen (∼18,000 genes). Among the potential candidates, we rediscovered KLF5, a known synthetic lethal partner for the tumor suppressor SMAD4 (31, 32), thus giving weight to our findings. We identified a new candidate gene known as Ras-related protein Rab-10 (RAB10). RAB10 is important for the proliferation of SMAD4-altered tumor cells. Overall, this study identifies RAB10 as a novel potential therapeutic target for SMAD4-negative tumors.

## Materials and Methods

### Cell Lines and Cell Biology Reagents

HT29, MIAPaCa-2, CFPAC1, and RKO were purchased from ATCC. DLD1 were obtained from Sigma, while SW620 and MDST8 were purchased from European Collection of Animal Cell Cultures and HUPT3 obtained from DSMZ. All cell lines were authenticated by short tandem repeat profiling analysis. They were routinely subjected to *Mycoplasma* testing and used up to 20 passages. HT29, MIAPaCa-2, CFPAC1, SW620, and MDST8 were maintained in DMEM (#11960, Life Technologies) supplemented with 10% FBS (#S181H-100, Biowest) and 2 mmol/L Glutamine (#AA29168, Life Technologies), while DLD1 were maintained in RPMI (#31870, Life Technologies) supplemented with 10% FBS and 2 mmol/L Glutamine and HUPT3 was cultured in MEMa 90% with 10% FBS, 2 mmol/L Glutamine; 1% nonessential amino acids; 1% sodium pyruvate. Doxycycline was purchased from Clontech (#631311) and dissolved in sterile water and filtered to make 1 mg/mL stock solutions. TGFβ recombinant human protein was purchased from Thermo Fisher Scientific (#CTP9211) and dissolved in sterile water to make 0.1 mg/mL stock solutions. Aliquots of the stock solutions were stored at −20°C.

### CellTiter-Glo Assay and Single Sphere Formation Assay

Cells were seeded in flat, U-bottom and in three-dimensional (3D) InSight (InSphero) 96-well plates in triplicate at a density of 500 cells per well. The following day, cells were cultured under doxycycline treatment for 10 days. Culture media was refreshed two times during the incubation period, keeping the final volume at 100 μL. Then, phase contrast pictures were taken (Incucyte ZOOM) to measure sphere diameters. To illustrate the sphere size differences, bright-field pictures were taken with the EnSight multimode plate reader (PerkinElmer). Finally, 100 μL of Celltiter Glo (Promega) was added to each well to determine effects of SMAD4 restoration on cell viability and proliferation.

### Antibodies and Immunoassay Using Capillary Electrophoresis

Whole-cell lysates were prepared using cell extraction buffer (FNN0011, Invitrogen) supplemented with protease and phosphatase inhibitors (Thermo Fisher Scientific, #1861282) and Basemuncher (BM0025, Expedeon). Protein extracts were prepared at 0.2 μg/μL, loaded, separated, and detected by the Sally Sue Simple Western system (ProteinSimple). The antibodies used in this study were used on Sally Sue at the following dilutions: anti-SMAD4 1:200 (#ab40759, Abcam), anti-GAPDH 1:200 (#2118, Cell Signaling Technology), anti-Cas9 1:200 (#14697, Cell Signaling Technology), anti-RAB10 1:100 (#8127, Cell Signaling Technology), anti SMAD3 1:100 (#9523), anti SMAD2 1:100 (#5339), and anti actin 1:100 (#4967).

### Cells Transfection and Plasmids

Inducible Cas9-expressing cell lines were obtained by transfecting parental cell lines with the pCM3586 neomycin-resistant plasmid using the lipofectamine 3000 Reagent (L3000015, Thermo Fisher Scientific) according to the manufacturer instructions. In the same way, inducible SMAD4 restoration was obtained by transfecting cells with the neomycin-resistant plasmid pSMAD4. After geneticin selection, clonal isolation was performed by limiting dilution.

### Assessment of SMAD Signaling Activity

Cells in which SMAD4 was restored with the inducible pSMAD4 plasmid, as well as control cells transfected with pCM3586 were transduced at a multiplicity of infection (MOI) of 32 TU/cell with the luciferase Cignal Lenti Reporter kit (CLS-017L, Qiagen). After puromycin selection, cells were seeded in 96-well plates in triplicate at a density of 1 × 10^4^ cells per well. The following day, cells were treated with doxycycline for 24 hours. Then TGFβ was added for 3 hours at a final concentration of 10 ng/mL. SMAD signaling activity was assessed by using the Bright-Glo Luciferase Assay system (Promega) according to the manufacturer instruction.

### *In Vivo* Tumorigenicity Assay

To assess the effects of SMAD4 restoration in SMAD4-deficient cell lines on tumorigenicity, parental cell lines as well as clones stably expressing a doxycycline-inducible SMAD4 gene (3 × 10^6^ cells/animal), were injected subcutaneously into the flank region of 8–10 weeks old female SCID mice (Charles River), weighing at least 18 g. Mice were randomized into three groups when tumors reached 150–300 mm^3^. Ten mice were left untreated, whereas 10 mice received vehicle (5% glucose #3058632 Lavoisier) and 10 additional mice received doxycycline (5 mg/mL in 5% glucose per bottle). Animal procedures were conducted in compliance with conditions established by the European Community (2010/63/EU Directive) and approved by the Sanofi Animal Care and Use Committee.

### Pooled Single-guide RNA Screen

The human whole-genome pooled CRISPR library used in this study was obtained from Thermo Fisher Scientific (#1715738 SO) as lentivirus particles with a titer of 29 × 10^8^ TU/mL. In the presence of 8 μg/mL of polybrene, 9 × 10^8^ cells were infected at an MOI of approximately 0.2 to ensure a unique integration of single-guide RNA (sgRNA) per cell, with a sgRNA coverage of × 1,000. Transduced cells were selected and expanded under 0.8–2 μg/mL puromycin for 15 days. After selection (T = 0), a reference sample was harvested and duplicate cultures were passaged with a seeding density of 2 × 10^8^ cells to maintain the library representation. Each cell line was passaged every 4–5 days until it reached 15 population doublings (PD). At each passage, the cumulative PD was calculated as follows:



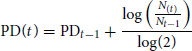



where *N*(*t*) is the number of cells counted at time (*t*) and *N*(*t* − 1) is the number of cells seeded at the previous time point, (*t* − 1). Cumulative population doublings were plotted against time. Doxycycline and puromycin were maintained all along the screen at 0.5–1 μg/mL and 0.8–1 μg/mL, respectively. Genomic DNA from harvested cell pools were extracted using the Blood and Tissue DNA Maxi kit (Qiagen, 13362). sgRNAs were amplified using the primers listed in Supplementary Table S1. More than 400 PCRs were performed for each sample to maintain the library coverage, with 3 μg of DNA per 50 μL PCR reaction. PCR products were purified and quantified using QuBit (Q32851), Ex-gel and Bioanalyser. sgRNAs were sequenced on a Illumina NextSeq and NovaSeq platform.

### Candidate Genes Identification, Data Processing, and Analysis

Sequencing reads were demultiplexed using the program “bcl2fastq.” Adapters were trimmed and the sgRNA sequences were mapped to the reference library using the Count function of MAGeCK-RRA (37, 38), giving normalized read counts per sample as output. Analyses were performed using the Test function of MAGeCK-RRA that rank genes based on their multiples targeting reagents, with PD15 samples as “test” samples and PD0 samples as baselines. For each PD0 versus PD15 comparison, the top 1,000 hits ranked by their MAGeCK negative score were compared trough cell lines, as well to an essential list of 684 genes published by Hart and colleagues (39). To keep only the most reliable hits, depleted genes appearing exclusively in the two SMAD4-negative cell lines and known as nonessential were considered as hits. We then used the public CRISPR screen database Depmap (https://depmap.org/portal/) to prioritize hits and validate further some of them.

### Colony Formation Assay

Unless specified otherwise, at day 0, colorectal cancer cells (DLD-1, SW620, and HT29) and pancreatic cancer cells (MIAPaCa-2) were seeded at 500 cells per well in 12-well plates. They were treated with doxycycline (0.25 μg/mL) at day 1 to induce the knockout (KO) and allowed to grow for 2 weeks before being washed with PBS and stained with crystal violet dye.

### Cell Proliferation Assay

Cells were seeded in T25 flasks at 3 × 10^5^ cells/flask. They were counted and split regularly under doxycycline treatment for 20 days. The total cell number reached after 20 days was estimated using the population doublings that were calculated all along the experiments at each harvest steps (see formula above). The proliferation percentage of each clone was calculated according to the proliferation percentage of the counterpart control cells having the nontargeting sgRNA.

### Analysis of RAB10 Expression and Copy-number Alteration of SMAD4 from TCGA

Patients data used in this study were obtained from the publicly available TCGA database (https://portal.gdc.cancer.gov/).

### Statistical Analysis

Data were presented as the mean ± SD. Statistical analyses were performed using GraphPad Prism (GraphPad Software). The difference between two groups was analyzed using an unpaired two-tailed Student *t* test. To perform multiple comparisons between tumors with different SMAD4 copy-number alteration (CNA; deep deletion, shallow deletion, diploid, gain), we performed a Kruskal–Wallis one-way ANOVA, followed by a *post hoc* Dunnett test to compare each group versus the “SMAD4 deep deletion” group (Fig. 5). *P* < 0.05 was considered statistically significant.

## Results

### SMAD4 Restoration Reestablished the TGFβ-induced Growth Inhibition in Cancer Cells *in Vitro* and in *Vivo*

**FIGURE 1:**
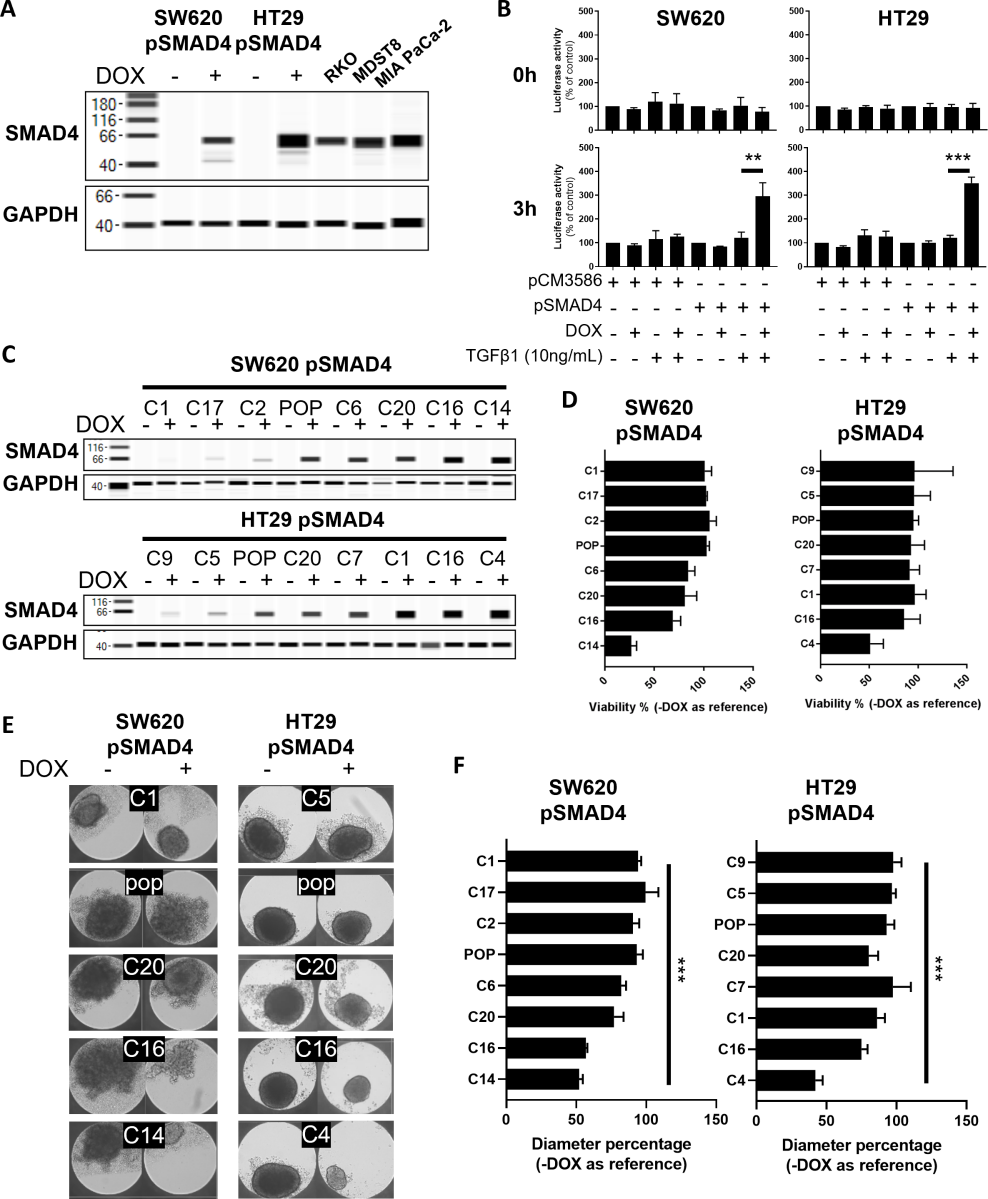
SMAD4-inducible restoration restores the TGFβ-induced growth inhibition in colorectal and pancreatic cancer cells. **A,** Protein level of SMAD4 was assessed by Western blot analysis in SMAD4-reexpressing HT29 and SW620 cells. RKO, MDST8 and MIAPaCa-2 were used as positive control. **B,** Luciferase reporter gene assay of TGFβ-induced SMAD promoter. SMAD4 functionality was assessed using a reporter assay in the reexpressing cell lines HT29 and SW620. Luciferase activity was normalized to control pCM3586 transfected cells, in absence of any treatment [*t* test performed on *n* = 3 (SW620); or *n* = 4 (HT29) independent experiments; *P* = 0.0000027 (HT29) *P* = 0.008 (SW620)]. Control plasmid pCM3586 leads to cells expressing Cas9 under doxycycline treatment. **C,** SMAD4 expression level was assessed in isolated clones from the HT29-pSMAD4 and SW620-pSMAD4 population (POP). **D,** SMAD4-reexpressing HT29-pSMAD4 and SW620-pSMAD4 clones and populations were seeded in 2D, in flat-bottom 96-well plates and cultured for 10 days with or without doxycycline. Proliferation was assessed using the CellTiter-Glo assay. Proliferation was normalized to untreated transfected cells (DOX−). *n* = 3 independent experiments. **E** and **F,** SMAD4-reexpressing HT29-pSMAD4 and SW620-pSMAD4 clones and populations were seeded in 96-well 3D plates and cultured for 10 days with or without doxycycline. Sphere diameters were measured. The diameter of the doxycycline-treated spheres reexpressing SMAD4 are represented with bar (percentage with untreated spheres taken as reference; percentage mean with SD, *n* = 3 independent experiments; *P* = 0.00025 for HT29 C4 vs. C9; *P* = 0.000027 for SW620 C1 vs. C14).

To check the dependence of tumor cell growth upon the alteration of SMAD4, we restored the tumor suppressor SMAD4 in HT29 and SW620 cell lines. Cells were transfected with a plasmid encoding inducible SMAD4, which allows the expression of SMAD4 under the control of an inducible Tet-On system (Supplementary Fig. S1A). As a control, we used the plasmid pCM3586 that expresses Cas9 under doxycycline treatment (Supplementary Fig. S1B). After one day of doxycycline exposure, protein lysates were collected and SMAD4 was detected by western blot. SMAD4 expression in HT29-pSMAD4 and SW620-pSMAD4 was comparable with endogenous expression levels determined in SMAD4-positive cell lines (Fig. 1A). To check whether the restored SMAD4 was functional, we introduced a luciferase reporter plasmid under the control of a CMV-SBE promoter in HT29-pSMAD4 and SW620-pSMAD4, as well as in their SMAD4-negative counterparts. Cells were seeded in 96-well plates and treated the following day with doxycycline for 24 hours. TGFβ was added at a final concentration of 10 ng/mL and SMAD signaling activity was assessed 0 and 3 hours after TGFβ exposure. SMAD activity was detected exclusively in the presence of SMAD4 after exposure to TGFβ, showing that the reintroduced SMAD4 in HT29-pSMAD4 and SW620-pSMAD4 was functional and restored response to the TGFβ pathway (Fig. 1B).

To assess the growth-suppressive effect of SMAD4 restoration, we isolated HT29-pSMAD4 and SW620-pSMAD4 clones expressing different levels of SMAD4 (Fig. 1C). We seeded the cells in two-dimensional (2D) into 96-well flat-bottom plates and performed CellTiter-Glo assays on these clones and on the population after 10 days of doxycycline exposure (Fig. 1D). We observed that cell proliferation was decreased in a SMAD4 expression level–dependent manner. The proliferation of clones displaying the highest levels of SMAD4 was reduced by 50% for HT29 and by 70% for SW620. To further show that SMAD4 restoration decreases cell proliferation, we performed 3D single sphere assays on the pSMAD4 clones. Five hundred cells were seeded into U-bottom wells and cultured with or without doxycycline for 10 days. Diameters of the spheres were measured and diameter percentages calculated with the untreated wells being used as reference (Fig. 1E and F). The diameter of clones displaying the highest levels of SMAD4 was reduced by about 50% in HT29 and SW620, indicating that SMAD4 restoration impairs sphere growth.

**FIGURE 2:**
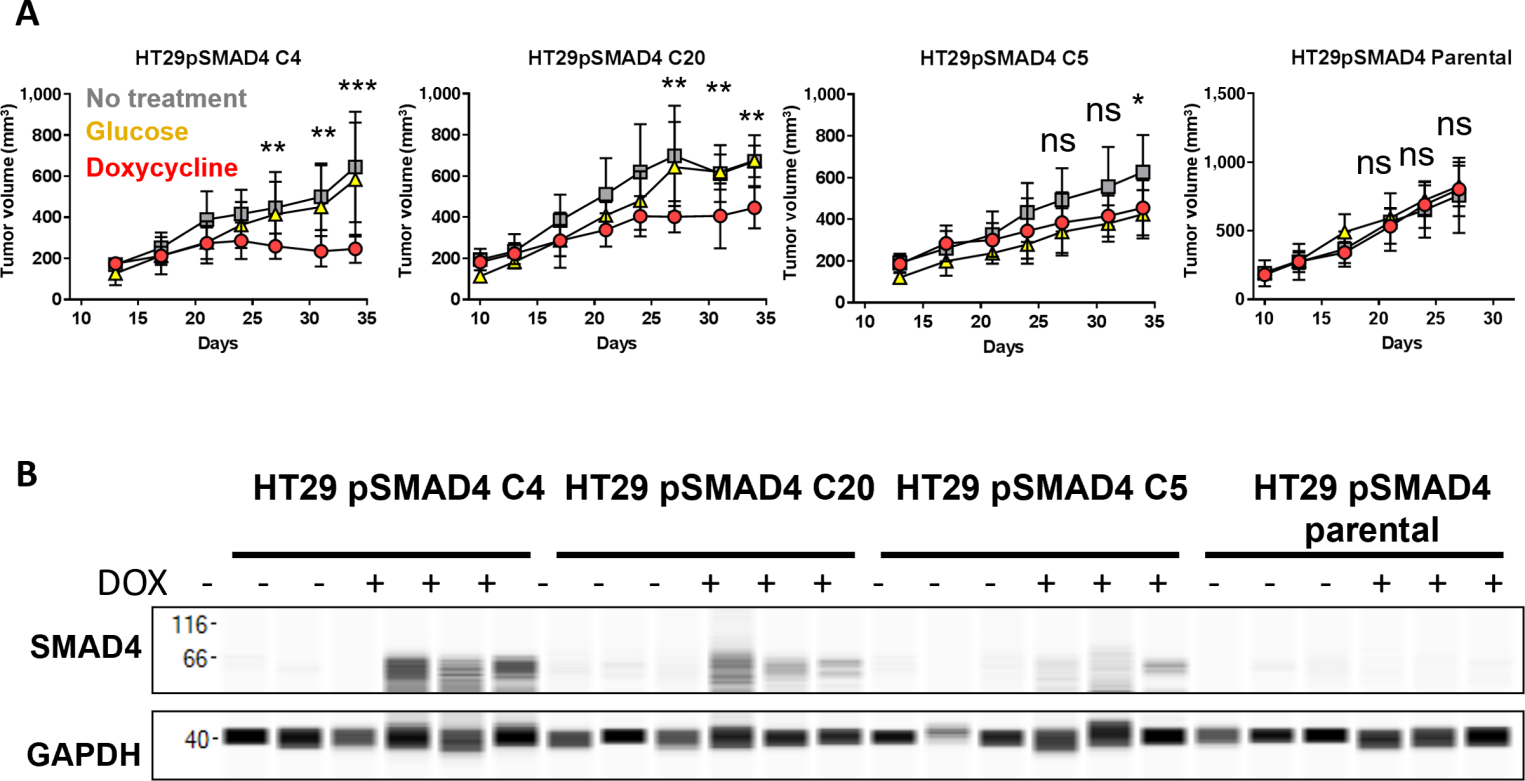
SMAD4-inducible restoration restores the TGFβ-induced growth inhibition of colorectal cancer cells *in vivo.***A,***In vivo* tumorigenicity assay. To assess the effects of SMAD4 restoration in SMAD4-deficient cell lines on tumorigenicity, parental cell line as well as clones stably expressing a doxycycline-inducible Smad4 were injected subcutaneously into the flank region of female SCID mice. Tumor volume over time is plotted. Square: no treatment; Triangle: Glucose treatment; Circle: Doxycycline treatment. Mean with SD (ns, *P* > 0.05; *, *P* ≤ 0.05; **, *P* ≤ 0.01; ***, *P* ≤ 0.001). **B,** At the end of the experiment, SMAD4-reexpressing HT29 tumors were harvested, frozen and a protein extraction was performed to assess the level of SMAD4 by Western blot analysis.

We then wanted to know whether SMAD4 restoration would result in a significant tumor volume reduction *in vivo*. We subcutaneously implanted HT29-pSMAD4 clones expressing different levels of SMAD4 in mice and allowed them to grow to approximately 150 mm^3^ and begun the doxycycline treatment orally. Tumor volumes were measured twice a week until tumors reached approximately 1,000 mm^3^ (Fig. 2A). To ensure that SMAD4 was properly expressed during the study, tumors were collected at the end of the experiment, and Western blots were performed on protein extracts to detect SMAD4 (Fig. 2B). Consistent with the *in vitro* results, SMAD4 restoration resulted in a significant decrease in tumor volumes *in vivo* and this positively correlated with SMAD4-expression levels.

Taken together, these results show that SMAD4 restoration through efficient transposon transfection restores TGFβ-induced growth inhibition in colorectal cancer cells, *in vitro* and *in vivo*. Thus, the ability of HT29 and SW620 to thrive remains in part, dependent on SMAD4 inactivation. These observations led us to hypothesize that cells that are dependent on SMAD4 loss would exhibit sensitivity to suppression of another gene and thus, could highlight synthetic lethal interactions involving SMAD4.

### Loss-of-function CRISPR Screen Identified Genes Involved in Synthetic Lethal Interaction with SMAD4 Loss

**FIGURE 3:**
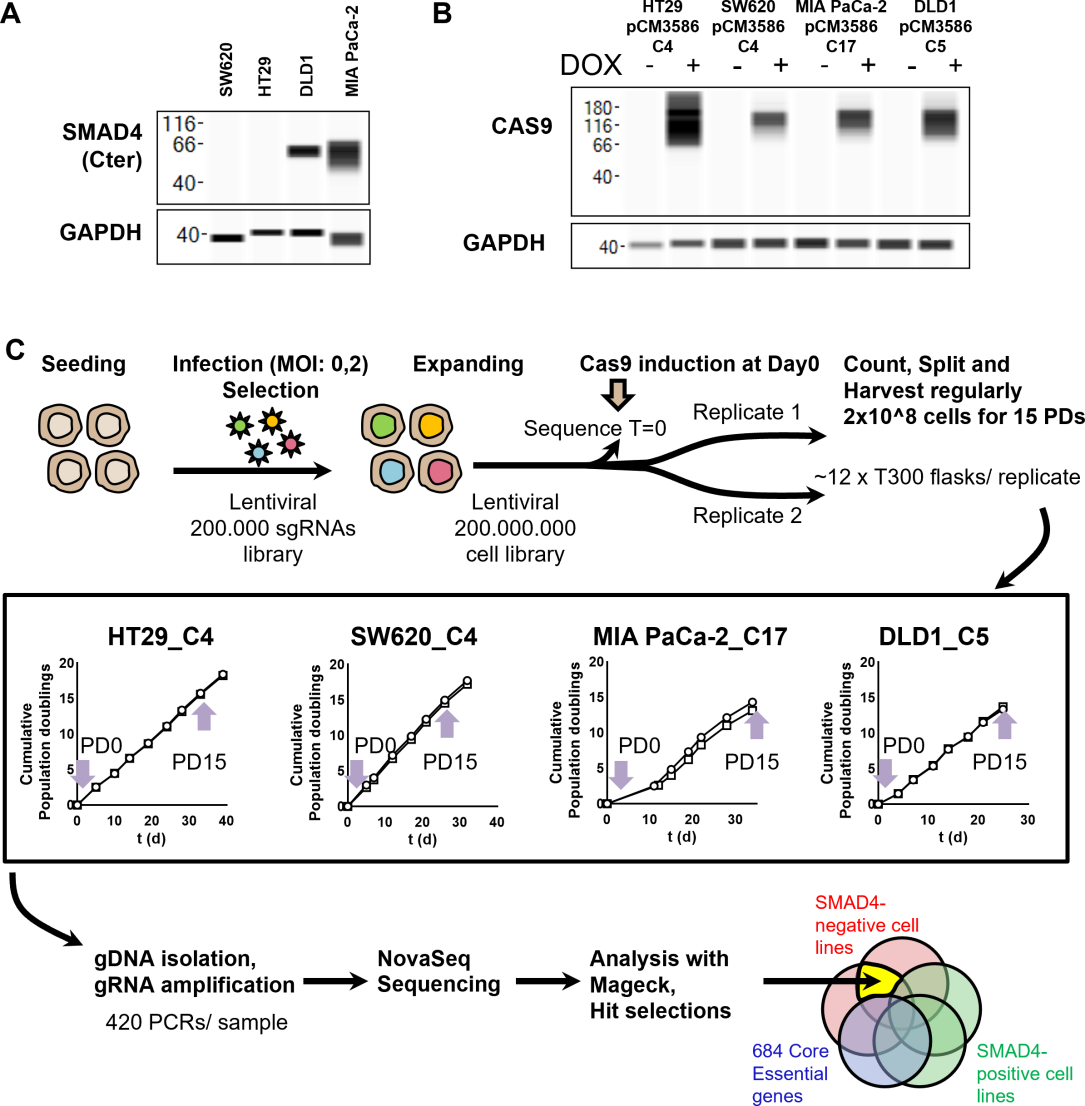
Construction and isolation of Cas9-expressing clones and workflow of our CRISPR screens. **A,** Levels of SMAD4 protein detected by the Sally Sue Simple Western system in the four cell lines HT29, SW620, DLD1, and MIAPaCa-2. **B,** Levels of Cas9 protein detected by the Sally Sue Simple Western system, in response to doxycycline treatment (exposure at 1 μg/mL for 3 days) in the four clones HT29, SW620, DLD1, and MIAPaCa-2 used in this study. **C,** Schematic representation of genome-wide CRISPR viability screens performed in HT29, SW620, DLD1, and MIAPaCa-2. Cells were infected at low MOI with a genome-wide library of sgRNAs. After selection, doxycycline was added to induce Cas9 and the KOs. Cells were regularly split and harvested, while keeping at least 200 million cells in culture to ensure a 1,000x library coverage. Cell growth curves of transduced cell lines following puromycin selection are plotted over time, for both replicates (circle and square). Arrows indicate samples that were harvested for sequencing at PD0 and PD15. sgRNA sequences were amplified and identified by NGS (NovaSeq). Enrichment/depletion of sgRNAS were assessed by comparing sgRNA counts at PD15 and PD0 using the Mageck analysis tool and by removing published core essential genes (Hart et al. 2017). PD, population doublings.

To identify genes whose loss of function decrease cell viability in absence of SMAD4, we performed a genome-wide CRISPR screen in two SMAD4-positive cell lines, MIAPaCa-2 and DLD1, as well as in two cell lines harboring SMAD4 mutations or deletions, HT29 and SW620 (Fig. 3A). These cell lines were first transfected with the plasmid pCM3586, to allow the inducible expression of Cas9 under doxycycline (Supplementary Fig. S1B). Screens were conducted on cellular clones expressing high levels of Cas9 obtained from limiting dilution (Fig. 3B).

We transduced HT29_Clone4, SW620_Clone4, MIAPaCa-2_Clone17, and DLD1_Clone5 with the Thermo Fisher Scientific pooled genome-wide library, which contains 174088 sgRNA constructs targeting 18,280 human genes (Fig. 3B). Infected cells were selected with puromycin and expanded for 15 days. After selection (T = 0), a reference sample was harvested (PD0) and duplicate cultures were passaged under doxycycline. Each cell line was passaged every 4–5 days until it reached 15 generations (population doubling PD15). At each step, no less than 200 million cells were harvested and kept in culture to ensure a 1,000x library coverage (Fig. 3B). For each cell line, genomic DNA was extracted from reference (PD0) and late samples (PD15). sgRNAs were amplified via 420 PCRs/sample to keep a high library representation and thus avoid any artificial dropout and ensure a good detection of depleted genes. Amplicons were then purified and sequenced via next-generation sequencing (Fig. 3C).

To assess the quality of our data, first we checked the representation of the sgRNA library at the beginning of the screen, by plotting the read count distribution at PD0 (Supplementary Fig. S2A.). On average, each sgRNA was read 60 to 375 times in each baseline PD0 samples. The DLD1 cell line had lower number of sgRNAs reads compared with other cell lines, probably due to its lower infectability. With 10 sgRNA per target gene, it was nevertheless considered as a sufficient representation. Second, we checked the coherence of the replicates by plotting principal component analysis on sgRNA counts across each screen sample (Supplementary Fig. S2B). Replicates of the screen showed high coherence in terms of the change in sgRNA representation. Then, additional QC data were plotted, showing that depletion of sgRNA is highly dependent of the Cas9 induction via doxycycline treatment rather than due to any uncontrolled evolution (Supplementary Fig. S2C). More than 80% of reads were mapped for each condition (Supplementary Fig. S2D), and for most of the samples, the Gini index, which reflects the degree of inequality in the distribution of sgRNAs counts was below 0.3 (Supplementary Fig. S2E). Of 174,085 sgRNAs, the number of zero-count sgRNAs did not exceed 35,000 except for DLD1 samples; and the number of sgRNAs with counts superior to 50 was above 88,000 (Supplementary Fig. S2F). These data are provided in Supplementary Table S2. Finally, we checked whether essential and nonessential genes have the expected behavior through samples. Taking common essential genes randomly (RPS15 and NAT10), as well as nonessential genes randomly (AFM and GRM5), we plotted the sgRNA counts targeting these genes in each sample (Supplementary Fig. S3). As expected, for all cell lines, sgRNA targeting core essential genes were depleted over time relative to sgRNA targeting nonessential genes. All in all, these QC provided confidence in the data that could be used for downstream analysis with MAGeCK. A table containing all the normalized sgRNA counts has been added in the Supplementary Data (Supplementary Table S3).

**FIGURE 4:**
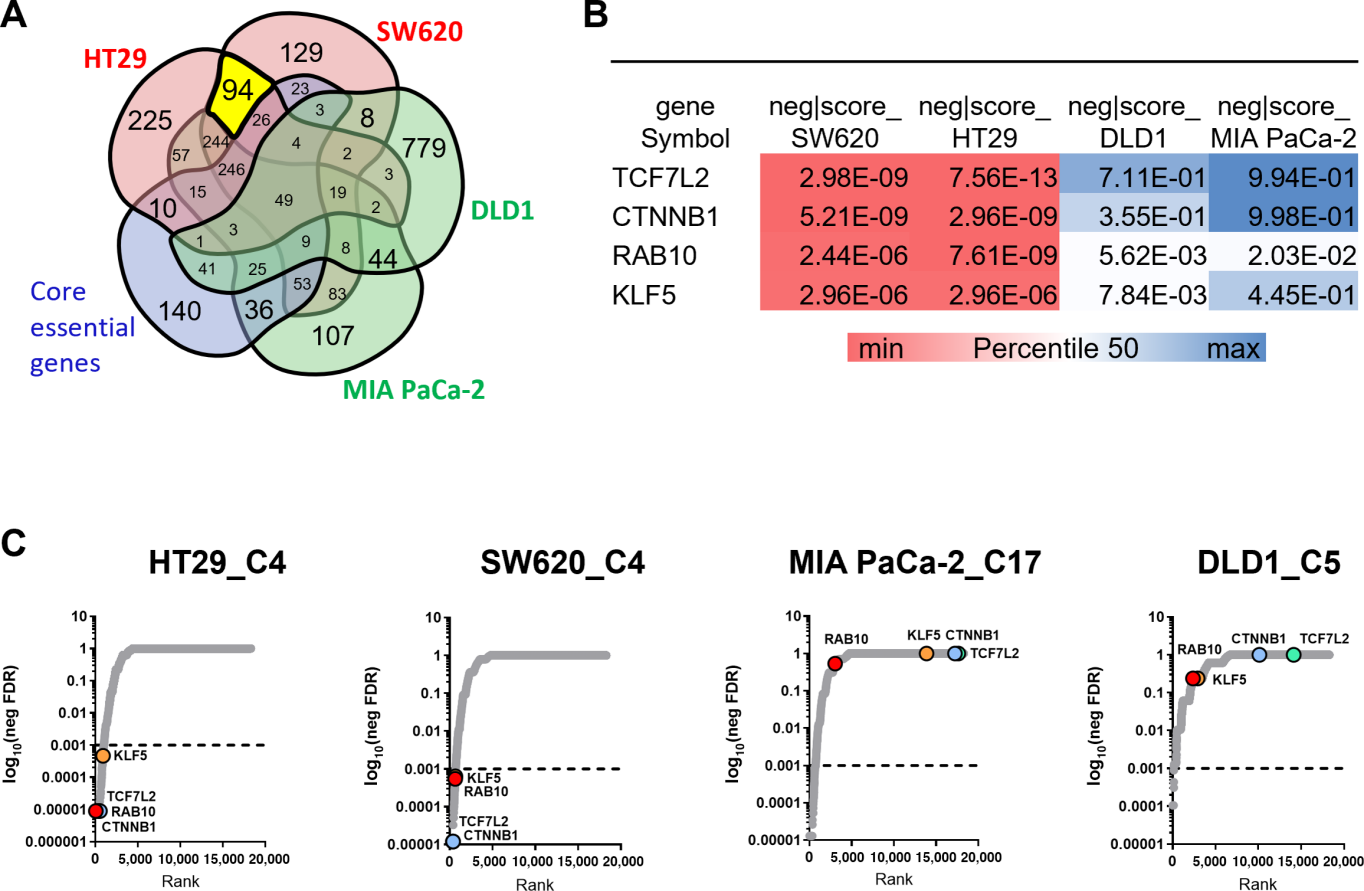
Identification of genes required for SMAD4-deficient cells viability via CRISPR/Cas9 viability screen. **A,** Identification of top candidates. Top 1,000 hits ranked by MAGeCK for each PD0 versus PD15 comparison were compared trough cell lines, as well to an essential list of genes published by Hart and colleagues (2017). A total of 94 genes that were not core-essential genes were found to be essential for the two SMAD4-negative cell lines. **B,** Heatmap of negative Mageck scores of the four selected top synthetic lethal genes. **C,** Depleted genes in MIAPaCa-2, DLD1, HT29, and SW620, ranked by their FDR from the MAGeCK analysis. The four top candidate genes are indicated with colored dots.

To compare the change in sgRNAs representation between PD0 and PD15 samples, we used the MAGeCK analysis tool (37). MAGeCK comparisons output and statistics have been put into the Supplementary Table S4 for all sgRNAs, and in Supplementary Table S5 for all genes. Identification of top candidates was performed as followed: for each PD0 versus PD15 comparison, the top 1,000 hits ranked by their MAGeCK negative score were compared trough cell lines, as well to an essential list of 684 genes published by Hart and colleagues (39). We found 94 nonessential genes to be depleted exclusively in the two SMAD4-negative cell lines (Fig. 4A; Supplementary Table S6). To further select potential synthetic lethal hits, the dependency score of the 94 genes and their correlations with SMAD4 CNA and SMAD4 mutation was assessed using the public 22Q2 Depmap values (https://depmap.org/portal/; Supplementary Fig. S4A). Four genes, RAB10, KLF5, TCF7L2, and CTNNB1 had gene essentiality strongly correlated with both SMAD4 mutation and SMAD4 CNA in various cancer cells (i.e., genes that are more essential when SMAD4 copy number is decreased or when SMAD4 is mutated, *P* values inferior to 1E-04). Because the scores and ranks of RAB10, KLF5, TCF7L2, and CTNNB1 from our CRISPR screen (Fig. 4B and C) were in line with databases, we decided to focus first and foremost on these genes. Notably, the Kruppel-like factor 5 (KLF5) had already emerged as a synthetic lethal partner for the tumor suppressor SMAD4 (31, 32) and the strong correlation between RAB10 essentiality and SMAD4 CNA observed in Depmap was similar to the one observed for KLF5 essentiality and SMAD4 CNA (Supplementary Fig. S4B). After a short validation screen based on colony formation assay using commercial sgRNAs targeting RAB10, KLF5, TCF7L2, and CTNNB1, in MIAPaCa-2 and HT29, it appeared that the KO of RAB10 showed a similar or even better an effect than the effect of KLF5 KO in the HT29 SMAD4-negative cell line (Supplementary Fig. S5).

**FIGURE 5:**
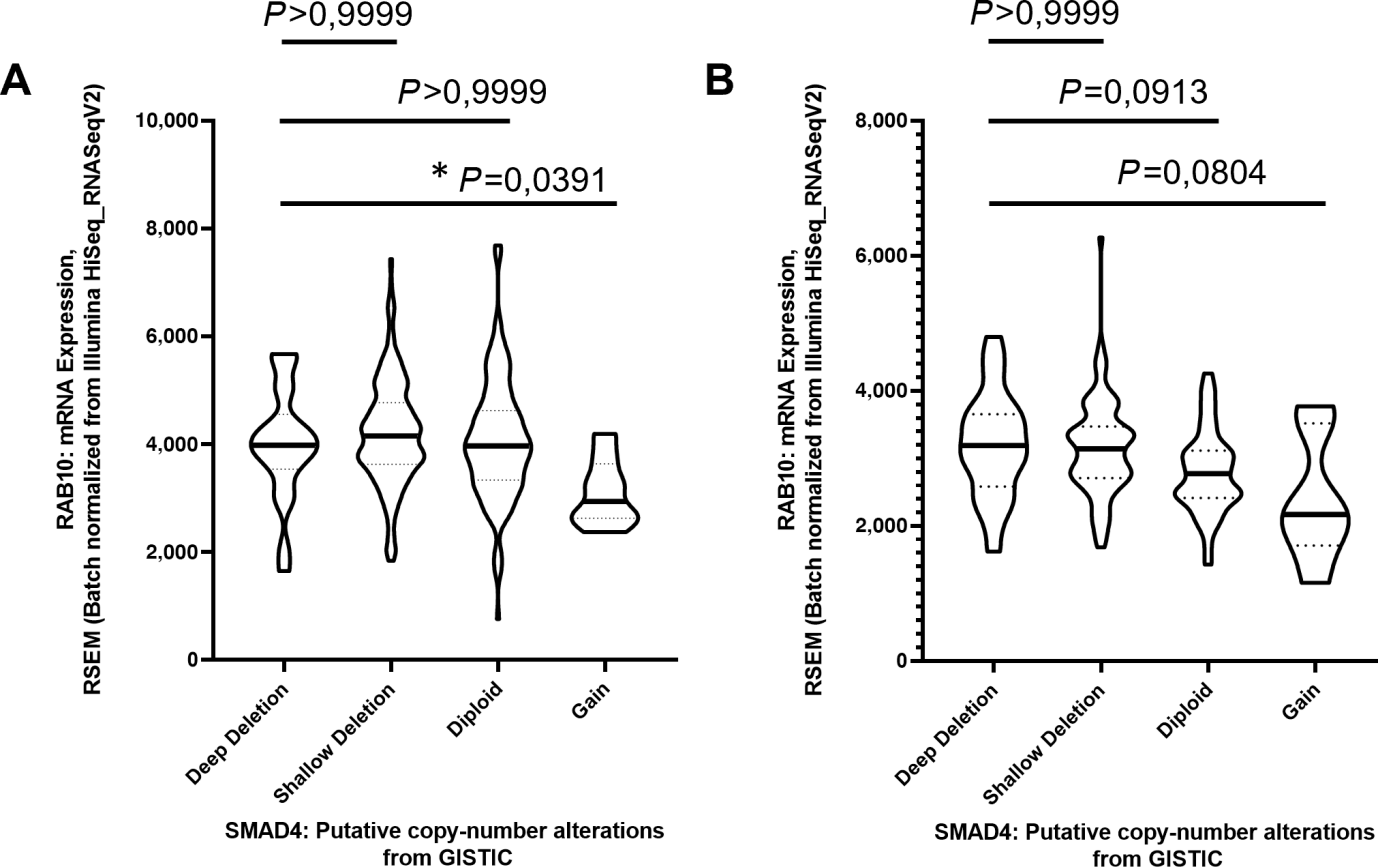
Association of the expression level of Rab10 with the CNA of SMAD4 in pancreatic and colorectal tumors. Violin plot showing the expression level of Rab10 relative to SMAD4 CNAs in colorectal cancer (TCGA, PanCancer Atlas, 524 patients, *, *P* < 0.05; **A**) and in pancreas cancers (pancreatic adenocarcinoma; TCGA, PanCancer Atlas, 168 patients; **B)**. For both panels, we performed a Kruskal–Wallis one-way ANOVA, followed by a *post hoc* Dunnett test to compare each group versus the “SMAD4 deep deletion” group.

RAB10 is a small GTPase that localizes to endocytic and exocytic compartments such as the endoplasmic reticulum, the Golgi apparatus, the endosomes/phagosomes, and the primary cilia. RAB10 is a key regulator of vesicular trafficking, including exocytic mechanism and endocytic processes (40). At the clinical level, TCGA analysis shows that RAB10 is significantly less expressed in colorectal tumors with a gain of SMAD4 as compared with tumors with deep deletion of SMAD4 (*, *P* = 0.0391 gain vs. deep deletion; Fig. 5A), suggesting that RAB10 expression is upregulated in absence of SMAD4. Comparable biological tendency was observed in pancreatic tumors, in which a decrease of RAB10 expression level seemed to be associated with a gain of SMAD4 (Fig. 5B). This observation reinforces the hypothesis of an essential regulatory role of RAB10 in absence of SMAD4. Therefore, for further validation experiments, we focused our study on RAB10.

### KO of RAB10 Decreases Cell Fitness of SMAD4-deficient Cell Lines

**FIGURE 6:**
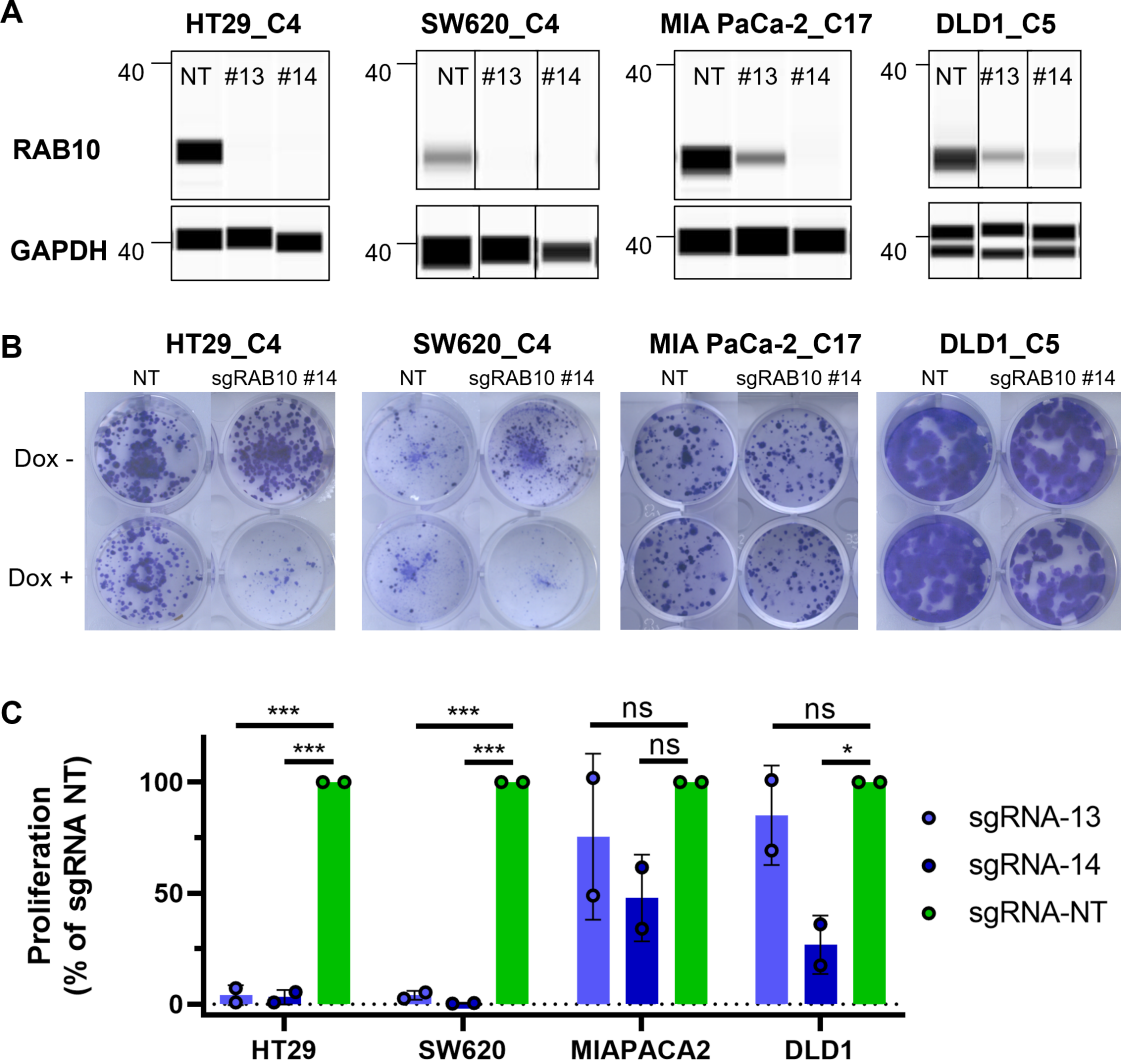
Validation of the screen showing that disruption of RAB10 decreases proliferation specifically of SMAD4-deficient colorectal and pancreatic cancer cells. **A,** RAB10 expression assessed with Western blot analysis in the HT29_C4, SW620_C4, DLD1_C5 and MIAPaCa-2_C17 clones expressing Cas9 and infected with sgRNA against RAB10 (sgRNA #13 and sgRNA #14) and control nontargeting sgRNA (sgRNA #NT). **B,** Colony formation assay performed on inducible Cas9-expressing clones that were infected with lentivirus-carrying two sgRNAs targeting RAB10 (sgRNA #13 and sgRNA #14), and one nontargeting sgRNA. Briefly, cells were seeded at 500 cells/well in 12-well plates and cells were treated with 0.5–1 μg/mL doxycycline at day 3 post seeding. Two weeks later, cells were stained with Cristal violet and plates were scanned. **C,** Proliferation assay. Cells were seeded in T25 flasks at 3 × 10^5^ cells/flask. They were counted and split regularly under doxycycline treatment for 20 days. The total cell number reached after 20 days was estimated using the population doublings that were calculated all along the experiments at each harvest steps. The proliferation percentage with SD of each clone is plotted according to the proliferation of the counterpart control cells having the nontargeting sgRNA (*N* = 2, *t* test sgRNA #13 vs. sgRNA #NT and sgRNA #14 vs. sgRNA #NT (*, *P* < 0.05; ***, *P* < 0.001; ns: nonsignificant). Note that the sgRNA used for the validation are distinct from the one present in the CRISPR screen library.

To further validate the screen results and confirm these findings, cells were infected with lentivirus-carrying two sgRNAs targeting RAB10 (sgRNA #13 and sgRNA #14), and one nontargeting sgRNA. These sgRNAs were different from those present in the CRISPR library (Supplementary Table S7). The KO efficiency of RAB10 was confirmed by Western blot analysis (Fig. 6A). To address whether RAB10 KO contributes to lethality of SMAD4-negative cell lines, we performed proliferation and colony formation assays. We observed that RAB10 depletion reduced cell proliferation specifically in SMAD4-negative cell lines in colony formation assays after 10 days (Fig. 6B). To further evaluate the impact of RAB10 depletion on SMAD4-negative cell line proliferation, we performed a proliferation assay, where the cells were cultured, counted, and split under doxycycline treatment for 20 days (Fig. 6C). While the proliferation of HT29 and SW620 was strongly reduced in absence of RAB10, MIAPaCa-2, and DLD1 were still able to proliferate. Similar results were obtained with two additional cell lines (Supplementary Fig. S6). The proliferation of the SMAD4-negative cell line CFPAC1 was impaired by RAB10 KO with a proliferation falling to 15%, while the SMAD4-positive HUPT3 were much less sensitive to RAB10 KO, giving weight to a potential synthetic lethal interaction between RAB10 and SMAD4.

**FIGURE 7:**
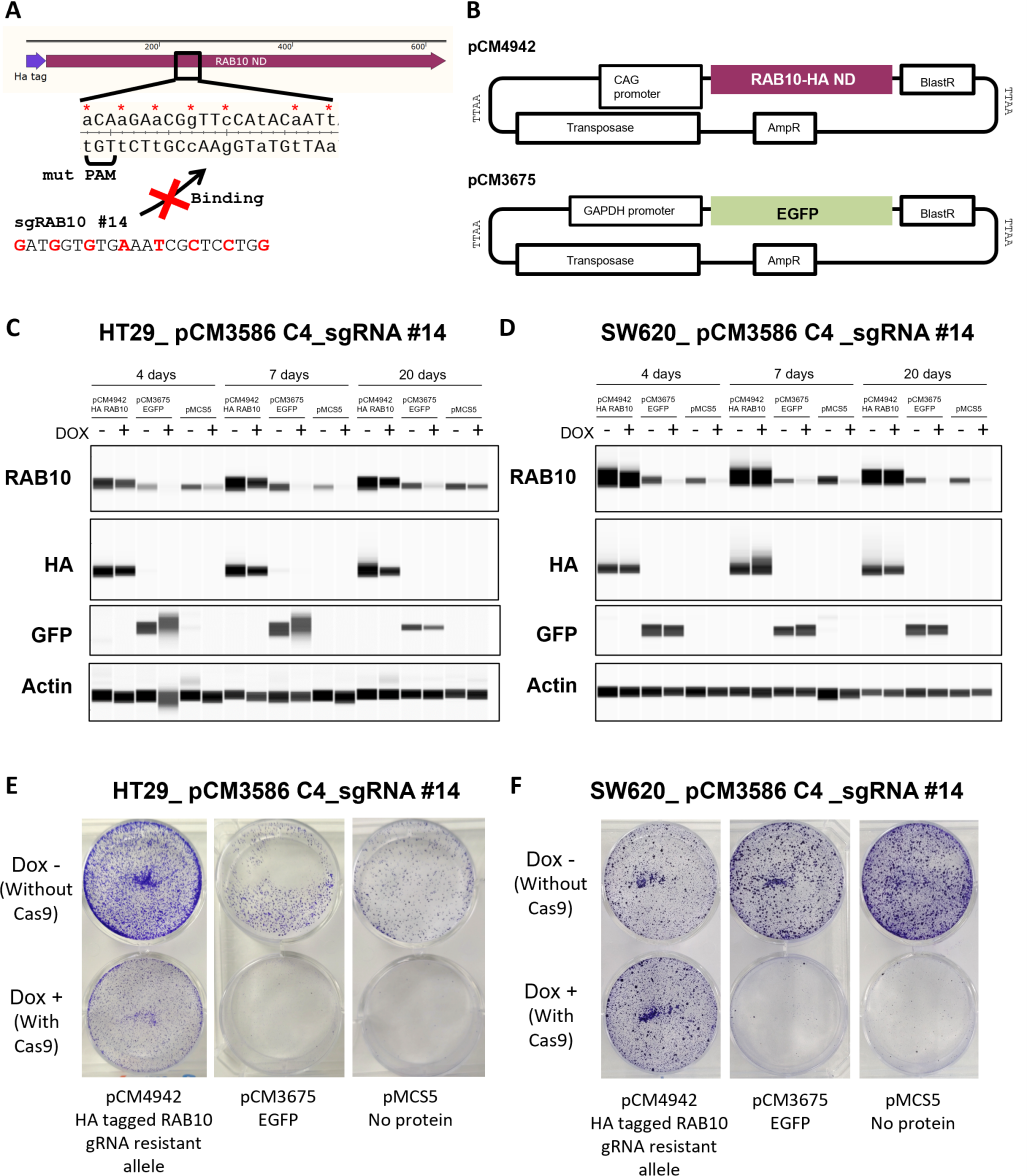
RAB10 is crucial for the proliferation of the SMAD4-negative SW620 and HT29 cell lines. **A,** Design of the ND HA-tagged RAB10 allele (HA-RAB10 ND), with seven mutations, including one mutation in the PAM sequence. **B,** Plasmid used to transfect SW620-pCM3586 C4 and HT29-pCM3586 C4. The plasmid pCM3675 contains EGFP, the plasmid pCM4942 contains the exogenous HA-RAB10 ND allele. Plasmid pMCS5 (not represented) is an empty counterpart. RAB10 expression assessed with Western blot analysis in the HT29_C4 (**C**) and in the SW620_C4 (**D**) clones expressing Cas9 under doxycycline treatment, infected with sgRNA against RAB10 (sgRNA #14), and transfected either with the empty plasmid pMCS5, EGFP (pCM3675), or with the HA-RAB10 ND allele (pCM4942). Colony formation assay performed on the inducible Cas9-expressing clones SW620-pCM3586 C4 (**E**) and HT29-pCM3586 C4 (**F**) that were infected with lentivirus-carrying sgRNAs targeting RAB10 (sgRNA #14), and transfected either with the empty plasmid pMCS5, EGFP (pCM3675), or with the HA-RAB10 ND allele (pCM4942). Cells were seeded at 5,000 cells per well in 6-well plates. They were treated with doxycycline (0.3 μg/mL) at day 1 to induce the KO and allowed to grow for 3 weeks before being washed with PBS and stained with crystal violet dye.

To counteract the susceptibility due to RAB10 KO in the setting of SMAD4 loss, we rescued RAB10 in the SMAD4-deficient cell lines SW620-pCM3586_C4 and HT29-pCM3586_C4 expressing sgRAB10 #14 (Fig. 7). Exogenous RAB10 cDNA had to be designed in a way that it is no longer recognized by the sgRNA already present in the cells (Fig. 7A). Seven silent mutations were introduced into the exogenous RAB10 gene. Six mutations were in the sequence that is normally recognized by the sgRAB10 #14, and one mutation was in the PAM sequence, thus making the recognition and cutting of the RAB10 gene by the sgRAB10 #14 impossible. This nondegradable (ND) allele version of RAB10 was linked to a HA tag, forming the “HA-RAB10 ND” allele.

Cells were transfected either with the control empty plasmid pMCS5, the control plasmid pCM3675 containing EGFP cDNA, or with the plasmid pCM4942, which harbors the exogenous HA-RAB10 ND allele (Fig. 7B). After having checked the proper expression via immunoblotting (Fig. 7C and D), colony formation assays were performed on cells that were treated with doxycycline to induce endogenous RAB10 KO. We confirmed that RAB10 KO is lethal for the SMAD4-negative SW620 and HT29 cells. We showed that cell viability was completely rescued with reintroduction of RAB10 in SW620 and HT29 cells having sgRAB10 #14 (Fig. 7E and F), confirming that the observed phenotype relied specifically on RAB10 KO, with no off-target effect.

All together, these data showed that RAB10 depletion contributed to decrease the cell fitness of SMAD4-deficient cells and that RAB10 might be a susceptibility gene in SMAD4-altered colorectal and pancreatic cancer cells.

## Discussion

The TGFβ signaling mediator SMAD4 is a key tumor suppressor whose loss or mutation is involved in colorectal and pancreatic tumorigenesis. This makes the context of SMAD4 loss an attractive target to identify synthetic lethal interactions and new therapeutic options.

In this study, we investigated synthetic lethal interactions involving SMAD4. As a prerequisite to find such synthetic lethal interactions, we first checked that our SMAD4-deficient cell lines were dependent on SMAD4 inactivation. We showed that the restoration of a functional Smad4 in SMAD4-defective cancer cells restored TGFβ signaling and decreased cell proliferation *in vitro*, particularly in 3D, and in proportion to their SMAD4 expression level. Consistent with previous reports, we also showed that the restoration of SMAD4 expression in SMAD4-defective cancer cells decreased *in vivo* tumorigenesis (41, 42).

Our CRISPR genome-wide loss-of-function screen was performed in four nonisogenic colorectal and pancreatic cancer cell lines, two SMAD4-negative and two SMAD4-positive cell lines. Using the Mageck ranking tools, the top 1,000 depleted genes were selected in each cell line. Once the core essential genes removed, our CRISPR screen led to the identification of 94 putative hits, which were refined to four top candidate genes with the help of the DepMap database. Among the candidates was KLF5, that has been shown to promote cancer cell proliferation in various cancers (26, 27). Our results are in line with previous reports showing that KLF5 is a susceptibility gene for SMAD4-negative cells (30–32). Our findings add to the evidence that KLF5 is a potentially interesting therapeutic target in SMAD4-negative colorectal and pancreatic cancers, even if transcription factor targeting is still challenging.

Other genes, such as AURKA and members of the BET, BRD2, and BRD4, have also recently been described as potentially synthetic lethal genes with SMAD4 loss (33, 34). In our screen, AURKA was not detected as essential for both HT29 and SW620, as it was not part of the top 1,000 ranked genes (rank of the negative score > 2,100/18,278 for all the screen cell lines). However, we did detect BRD2 as essential for the SMAD4-negative cell line SW620 (rank of the negative score = 470), but BRD2 also came out as essential for the control SMAD4-positive cell line MIAPaCa-2 (rank of the negative score = 296) and not essential for the other SMAD4-negative cell line HT29 (rank of the negative score = 13,675).

Loss of ME2 in the SMAD4 locus has been shown to create a cancer-specific metabolic vulnerability upon targeting of its paralogous isoform ME3 in pancreatic ductal adenocarcinoma lacking SMAD4 (35). This “collateral lethality” mediated by ME3 deletion was not found in our screen (rank of the negative score > 7,000/18,278 for all the screen cell lines) and this was expected as neither SW620 nor HT29 have a SMAD4 biallelic deletion that would lead to a collateral ME3 deletion (HT29: CNA = 1 and SMAD4 mutation Q311*; SW620: SMAD4 CNA = 1 and SMAD4 mutation IVS7 + 5G>C).

Interestingly, we identified several genes that have not yet been studied as synthetic lethal with SMAD4. Considering the CRISPR screen database DepMap, as well as clinical data, and due to early convincing findings emerging from a short screen based on colony formation assay, we focused the validation of our screen on the RAS-related protein RAB10. Our data show that RAB10 KO decreases proliferation preferentially of SMAD4-deficient colorectal and pancreatic cancer cell lines. This specific susceptibility suggests that RAB10 becomes important in the absence of SMAD4. Supporting this view, RAB10 expression is increased in colorectal tumors that have deep deletions of SMAD4 as compared with tumors with gain of SMAD4. RAB10 is a GTPase that serves as a regulator of intracellular vesicle trafficking (40). Like other small GTPases in the Ras superfamily, Rabs show high affinity for guanine nucleotides GTP and GDP (Kd in the nanomolar range; ref. 43). RAB10 cycles between an inactive GDP-bound form and an active GTP-bound form that can recruit to the membrane different downstream effectors responsible for exocytic polarized targeting and endocytic recycling events. Given the numerous roles of RAB10 protein on cellular trafficking, its link to SMAD4 biology and tumorigenesis remains to be fully determined.

Our preliminary results suggest that RAB10 is more probably a susceptibility gene that is not directly connected to SMAD4. In our *in vitro* experiments, SMAD4 reintroduction in the SW620 and HT29-deficient cell lines had no impact on RAB10 protein level. Conversely, RAB10 depletion in HT29 and SW620 cell lines does not impact SMAD3 and SMAD2 protein level (Supplementary Fig. S7). In addition, upon SMAD4 reintroduction in HT29 and SW620, we observed a clear morphologic change of the cells that turned into less cohesive and elongated cells, but we did not observe a clear RAB10 localization change (Supplementary Fig. S8). This would suggest that RAB10 is not directly connected to SMAD signaling.

One hypothesis to test in future work would be the link between SMAD4 loss, cell trafficking/recycling, and epithelial–mesenchymal transition (EMT) program. EMT is a crucial process in cancer metastasis. EMT is a biological process where epithelial cells lose their characteristics to acquire a spindle-cell phenotype, stemness characteristics, and increased motility. At the molecular level, EMT is frequently characterized by expression and localization of markers, such as β-catenin, Snail, Slug, Twist, Nestin, and Vimentin. The TGFβ pathway is known to play a role in EMT (44). SMAD4 has been suggested as a central component of EMT transition in human colorectal cancer (45), but in absence of SMAD4, alternative factors may take over the gene regulatory functions of SMAD4 to drive EMT (46). In this vein, it has been shown that in a SMAD4-null context, SMAD2 and SMAD3 transduce TGFβ-driven cell migratory and invasive properties via the initiation of a SMAD4-independent “collective migration” transcriptional program (47). Dynamic assembly of cytoskeletal structures on the leading edge of motile cells requires precise spatial and temporal control of protein trafficking. It could be interesting to test whether the RAB10 essentiality in absence of SMAD4 could be explained by a SMAD4-independent migratory behavior, which is not supported by the trafficking machinery in absence of RAB10, leading to cell death. In addition to Smad-dependent signaling, TGFβ also initiates non-Smad signaling from TβRII/TβRI complexes, leading to activation of pathways involving receptor tyrosine kinase signaling, such as PI3K/Akt, Erk, and p38 MAPK, and Rho-GTPases pathways (48–50). As endocytosis mediates receptor degradation and plays an important role in controlling the amount of receptors on the plasma membrane (51), it would be interesting to test whether inhibiting RAB10-dependent cellular trafficking would impair the localization of the TβRII/TβRI complex itself, or of other key tyrosine kinase receptors, whose disruption would impair a SMAD4-independent EMT, and lead to cell death.

In summary, our work identifies RAB10 as a potentially synthetic lethal gene in SMAD4-negative colorectal and pancreatic cancer cells. Further investigation is necessary to shed light on the mechanism by which RAB10 inhibition decreases cell proliferation of SMAD4-negative cells. To determine whether targeting RAB10 in SMAD4-deficient colorectal and pancreatic cancers would exhibit a therapeutic value in the long run, further efforts are also necessary to develop inhibitors specifically targeting RAB10. GTPases are challenging therapeutic targets, often considered as “undruggable.” However, there have been advances in directly targeted drugs for GTPase such as the mutated G12C and G12D KRAS (52, 53), opening up promising prospects for drugging undruggable pockets.

## Supplementary Material

Table S1 library preparation primers

Table S2 QCsummary

Table S3 sgRNAcounts

Table S4 sgRNAscoreMageckComparison

Table S5 Gene_scoreMageckComparison

Table S6 94 Putative synthetic lethal genes identified in the screen

Table S7 sgRNAs against RAB10

Supplementary Figure 1 Map of the plasmids to express inducible SMAD4 and Cas9

Supplementary Figure 2 S2. Quality check of the CRISPR screen samples.

Supplementary Figure 3 Behavior of sgRNAs targeting essential and nonessential genes in the screen samples.

Supplementary Figure 4 RAB10 essentiality in cells having altered SMAD4 is confirmed by the in vitro CRISPR screen database DepMap

Supplementary Figure 5 Short validation screen in plate to validate the top 4 synthetic lethal gene candidates

Supplementary Figure 6 Validation of RAB10 susceptibility in two additional cell lines.

Supplementary Figure 7 Proteins level assessment by western blotting

Supplementary Figure 8 Determination of RAB10 localization
