# Supplementary material for: Genome-wide CRISPR Screen Reveals RAB10 as a Synthetic Lethal Gene in Colorectal and Pancreatic Cancers Carrying SMAD4 Loss
Source: Cancer Res Commun. 2023 May 4;3(5):780–92. doi: 10.1158/2767-9764.CRC-22-0301 (PMC10158796; doi:10.1158/2767-9764.CRC-22-0301)
Supplement: Supplementary Figure 1 — Map of the plasmids to express inducible SMAD4 and Cas9 [file crc-22-0301-s08.pdf]

Figure S1

A

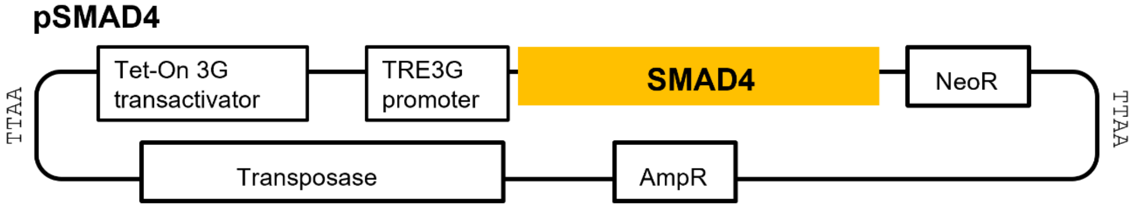
pSMAD4
TTAA
Tet-On 3G transactivator
TRE3G promoter
SMAD4
NeoR
TTAA
Transposase
AmpR

B

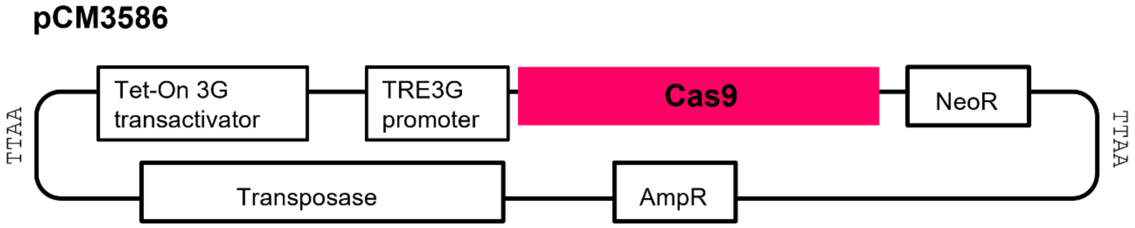
pCM3586
TTAA
Tet-On 3G transactivator
TRE3G promoter
Cas9
NeoR
TTAA
Transposase
AmpR

Figure S1: Map of the plasmids to express inducible SMAD4 and Cas9

A, Plasmid pSMAD4 allows the inducible expression of SMAD4. **B**, Plasmid pCM3586 allows the inducible expression of CAS9. Both plasmids contain a Neomycin resistant cassette (NeoR), as well as a doxycycline inducible Cas9 or SMAD4 cassette, under the control of a tetracycline response element TRE3G promoter, and a Tet-On 3G transactivator cassette.
