# Supplementary material for: Genome-wide CRISPR Screen Reveals RAB10 as a Synthetic Lethal Gene in Colorectal and Pancreatic Cancers Carrying SMAD4 Loss
Source: Cancer Res Commun. 2023 May 4;3(5):780–92. doi: 10.1158/2767-9764.CRC-22-0301 (PMC10158796; doi:10.1158/2767-9764.CRC-22-0301)
Supplement: Supplementary Figure 2 — S2. Quality check of the CRISPR screen samples. [file crc-22-0301-s09.pdf]

Figure S2

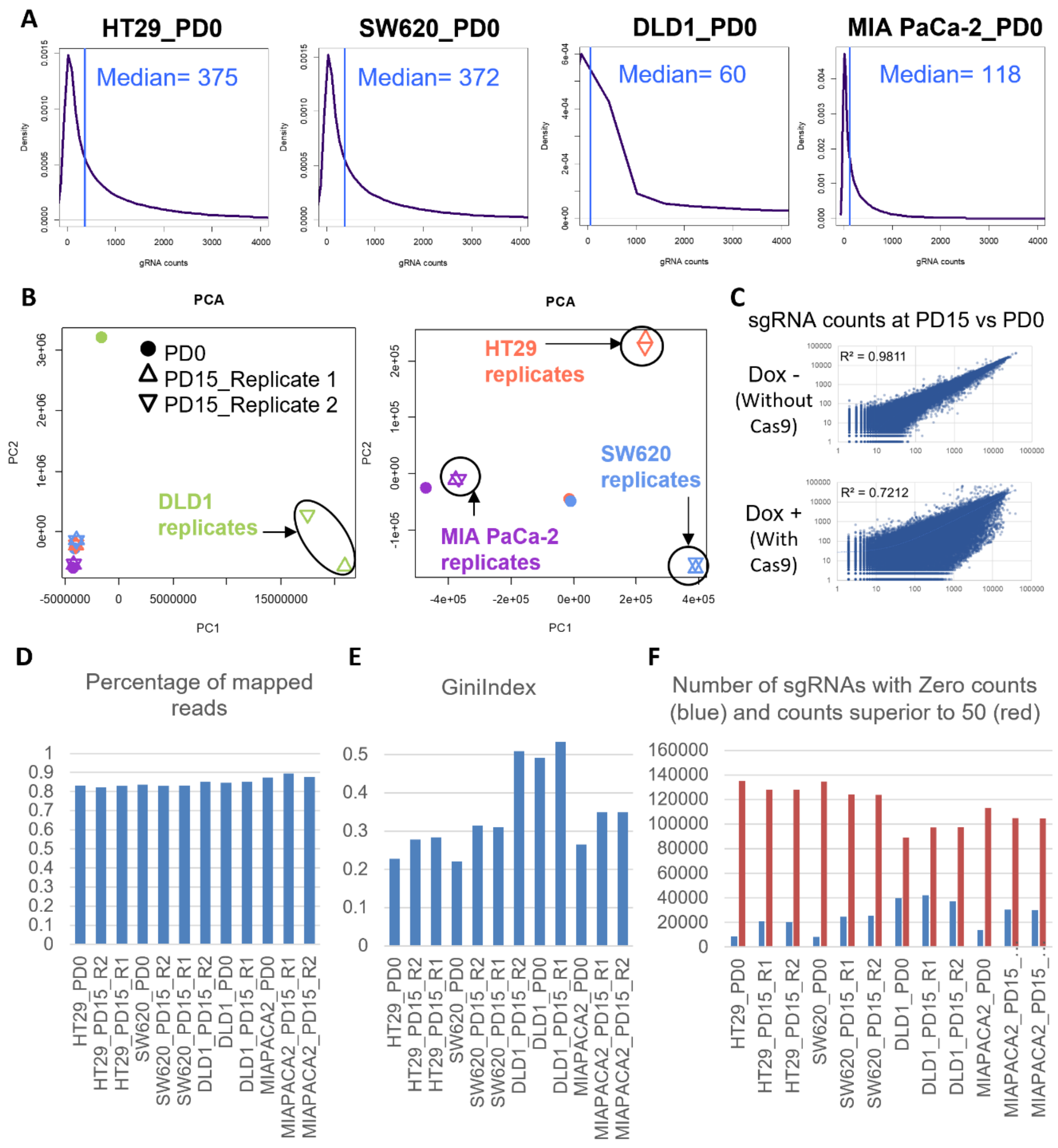

A
HT29_PD0
Median= 375
SW620_PD0
Median= 372
DLD1_PD0
Median= 60
MIA PaCa-2_PD0
Median= 118
Density
gRNA counts
B
PCA
PD0
PD15_Replicate 1
PD15_Replicate 2
DLD1 replicates
HT29 replicates
SW620 replicates
MIA PaCa-2 replicates
PC1
PC2
C
sgRNA counts at PD15 vs PD0
Dox - (Without Cas9)
R² = 0.9811
Dox + (With Cas9)
R² = 0.7212
D
Percentage of mapped reads
E
GiniIndex
F
Number of sgRNAs with Zero counts (blue) and counts superior to 50 (red)

Figure S2: Quality check of the CRISPR screen samples. A, Distribution of sgRNA counts in PD0 baseline samples. On average, there was 60 to 375 sequencing reads per sgRNA in each baseline PD0 samples. With 10 sgRNA per target gene, it was considered a sufficient representation. **B,** PCA on sgRNA counts across each screen samples. Replicates of the screen showed high coherence in terms of the change in sgRNA representation. **C,** sgRNA counts at PD15 vs PD0, from the HT29 screen performed with or without doxycycline (i.e. with or without Cas9 induction). Depletions are exclusively observed under doxycycline treatment. **D,** Bar plot of the percentage of mapped reads. **E,** Bar plot of the Gini index. **F,** Bar plot of the number of zero-count sgRNAs and sgRNAs counts superior to 50 through the screen samples. PD0: population doubling 0; PD15: population doubling 15; R1: replicate 1; R2: replicate 2.
