# Supplementary material for: Genome-wide CRISPR Screen Reveals RAB10 as a Synthetic Lethal Gene in Colorectal and Pancreatic Cancers Carrying SMAD4 Loss
Source: Cancer Res Commun. 2023 May 4;3(5):780–92. doi: 10.1158/2767-9764.CRC-22-0301 (PMC10158796; doi:10.1158/2767-9764.CRC-22-0301)
Supplement: Supplementary Figure 3 — Behavior of sgRNAs targeting essential and nonessential genes in the screen samples. [file crc-22-0301-s10.pdf]

Figure S3

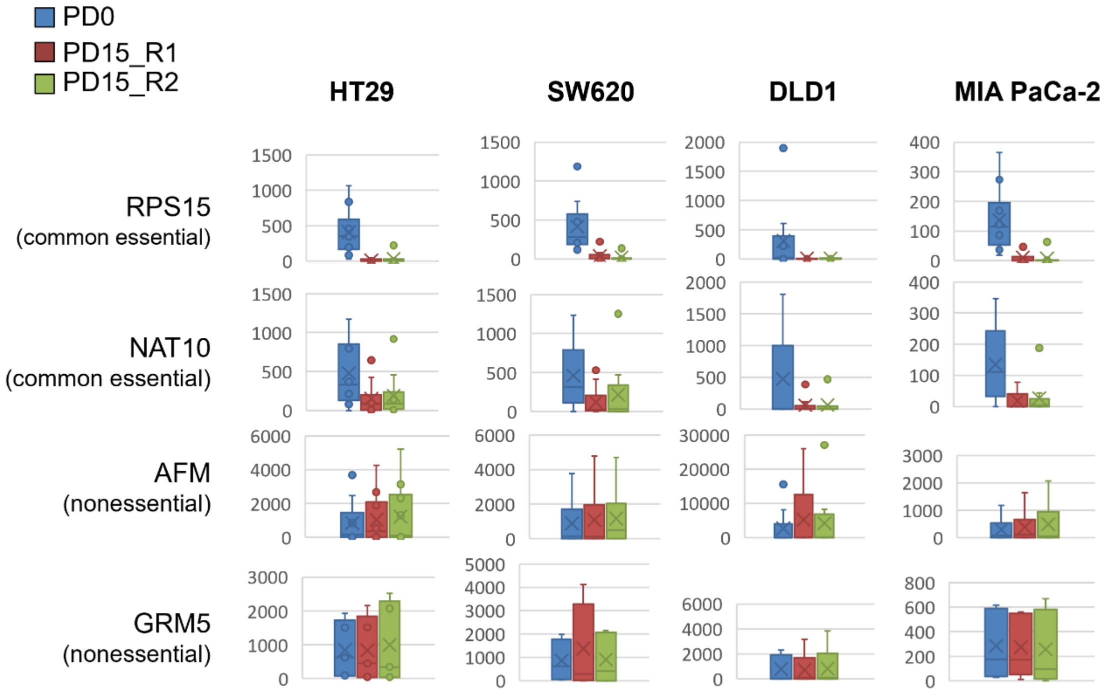

PD0
PD15_R1
PD15_R2
HT29
SW620
DLD1
MIA PaCa-2
RPS15
(common essential)
NAT10
(common essential)
AFM
(nonessential)
GRM5
(nonessential)

Figure S3: Behavior of sgRNAs targeting essential and nonessential genes in the screen samples. Box plots of sgRNA counts targeting randomly chosen essential genes (RPS15 and NAT10) and nonessential genes (AFM and GRM5) in each screen samples. PD0: population doubling 0; PD15: population doubling 15; R1: replicate 1; R2: replicate 2.
