# Supplementary material for: Genome-wide CRISPR Screen Reveals RAB10 as a Synthetic Lethal Gene in Colorectal and Pancreatic Cancers Carrying SMAD4 Loss
Source: Cancer Res Commun. 2023 May 4;3(5):780–92. doi: 10.1158/2767-9764.CRC-22-0301 (PMC10158796; doi:10.1158/2767-9764.CRC-22-0301)
Supplement: Supplementary Figure 4 — RAB10 essentiality in cells having altered SMAD4 is confirmed by the in vitro CRISPR screen database DepMap [file crc-22-0301-s11.pdf]

Figure S4

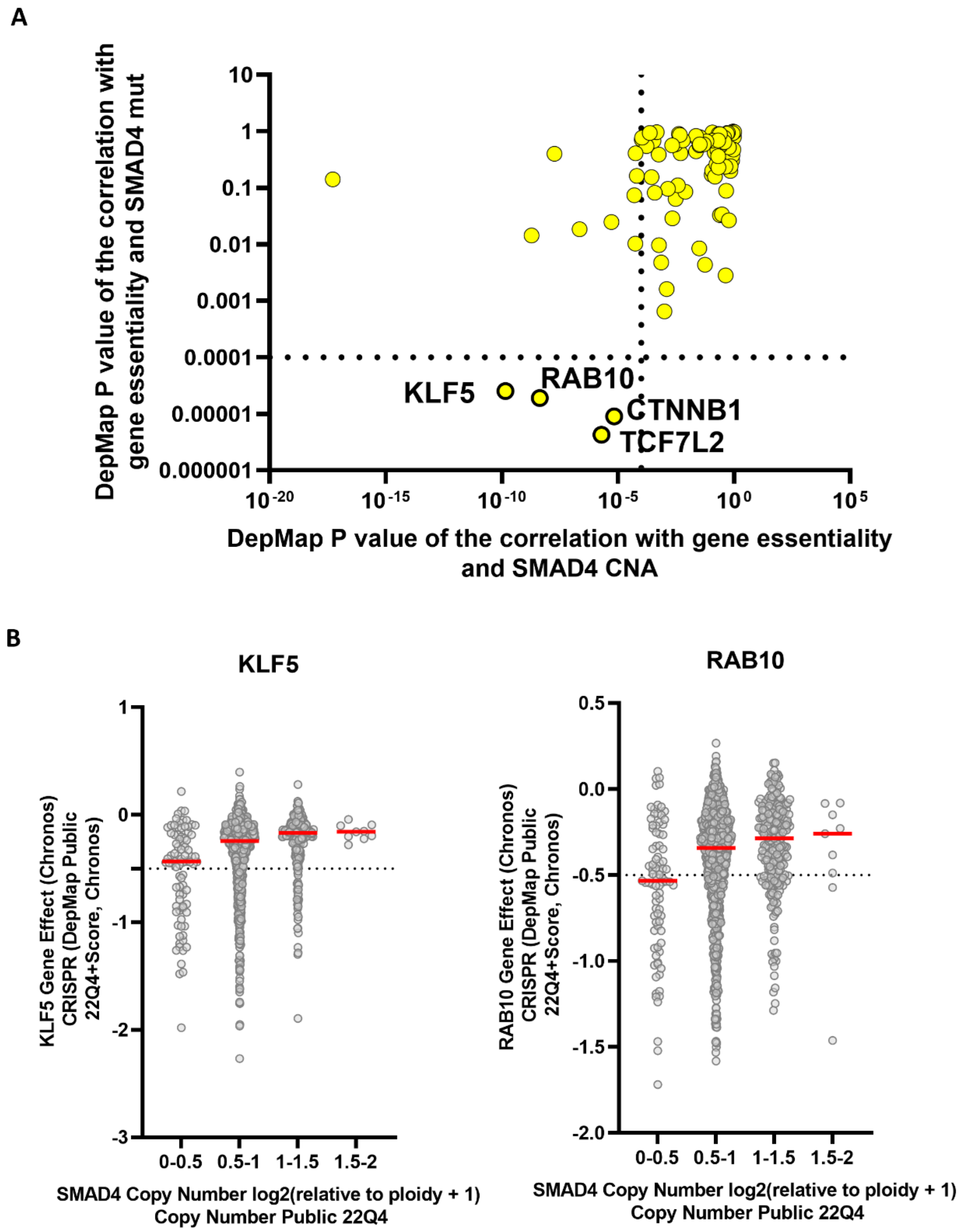
A
DepMap P value of the correlation with gene essentiality and SMAD4 mut
10
1
0.1
0.01
0.001
0.0001
0.00001
0.000001
KLF5
RAB10
CTNNB1
TCF7L2
10 -20
10 -15
10 -10
10 -5
10 0
10 5
DepMap P value of the correlation with gene essentiality and SMAD4 CNA
B
KLF5
KLF5 Gene Effect (Chronos) CRISPR (DepMap Public 22Q4+Score, Chronos)
1
0
-1
-2
-3
0-0.5
0.5-1
1-1.5
1.5-2
SMAD4 Copy Number log2(relative to ploidy + 1) Copy Number Public 22Q4
RAB10
RAB10 Gene Effect (Chronos) CRISPR (DepMap Public 22Q4+Score, Chronos)
0.5
0.0
-0.5
-1.0
-1.5
-2.0
0-0.5
0.5-1
1-1.5
1.5-2
SMAD4 Copy Number log2(relative to ploidy + 1) Copy Number Public 22Q4

Figure S4: RAB10 essentiality in cells having altered SMAD4 is confirmed by the in vitro CRISPR screen database DepMap

A, Dot plot of the P value of the correlation between the essentiality of each of the 94 hits and SMAD4 status (CNA and mutation) from the DepMap database. KLF5, RAB10, CTNNB1 and TCF7L2 essentiality is strongly correlated to both SMAD4 CNA and SMAD4 mutation (P < 10-4). CNA: Copy number alteration; mut : mutation. **B,** Dot plot of KLF5 and RAB10 essentiality score from the DepMap database according to SMAD4 copy number. Median are displayed in red. A Chronos score below 0.5-0.6 is considered to reflect gene essentiality.
