# Supplementary material for: Genome-wide CRISPR Screen Reveals RAB10 as a Synthetic Lethal Gene in Colorectal and Pancreatic Cancers Carrying SMAD4 Loss
Source: Cancer Res Commun. 2023 May 4;3(5):780–92. doi: 10.1158/2767-9764.CRC-22-0301 (PMC10158796; doi:10.1158/2767-9764.CRC-22-0301)
Supplement: Supplementary Figure 5 — Short validation screen in plate to validate the top 4 synthetic lethal gene candidates [file crc-22-0301-s12.pdf]

Figure S5

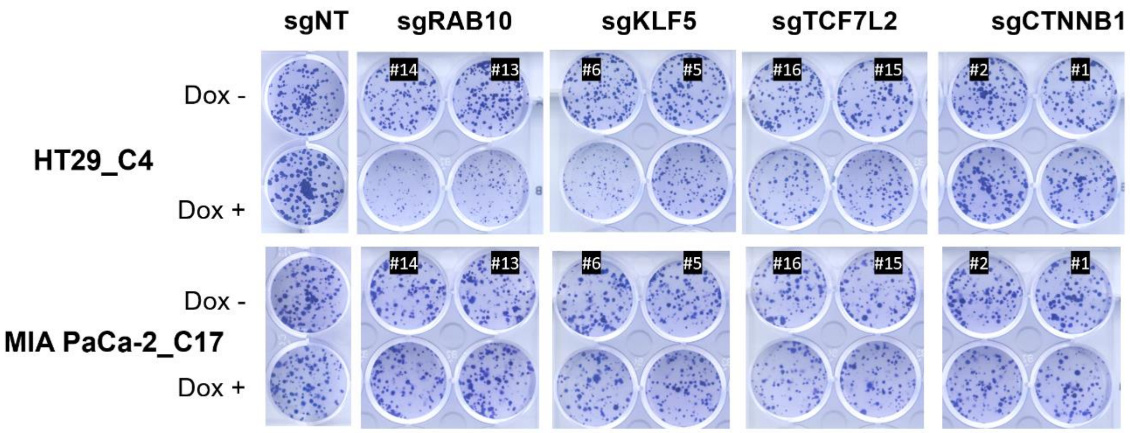

sgNT
sgRAB10
sgKLF5
sgTCF7L2
sgCTNNB1
HT29_C4
Dox -
Dox +
MIA PaCa-2_C17
Dox -
Dox +
#14
#13
#6
#5
#16
#15
#2
#1

Figure S5: Short validation screen in plate to validate the top 4 synthetic lethal gene candidates

Colony formation assay performed on inducible Cas9-expressing clones, HT29_C4 and MIAPaCa-2_C17 that were infected with lentivirus-carrying two sgRNAs targeting the four genes of interest, and one non-targeting sgRNA. Only the RAB10 KO was checked. Briefly, cells were seeded at 500 cells/well in 12-well plates and cells were treated with 0.5-1 µg/mL doxycycline at day 3 post- seeding. Two weeks later, cells were stained with Cristal violet and plates were scanned.
