# Supplementary material for: Genome-wide CRISPR Screen Reveals RAB10 as a Synthetic Lethal Gene in Colorectal and Pancreatic Cancers Carrying SMAD4 Loss
Source: Cancer Res Commun. 2023 May 4;3(5):780–92. doi: 10.1158/2767-9764.CRC-22-0301 (PMC10158796; doi:10.1158/2767-9764.CRC-22-0301)
Supplement: Supplementary Figure 6 — Validation of RAB10 susceptibility in two additional cell lines. [file crc-22-0301-s13.pdf]

Figure S6

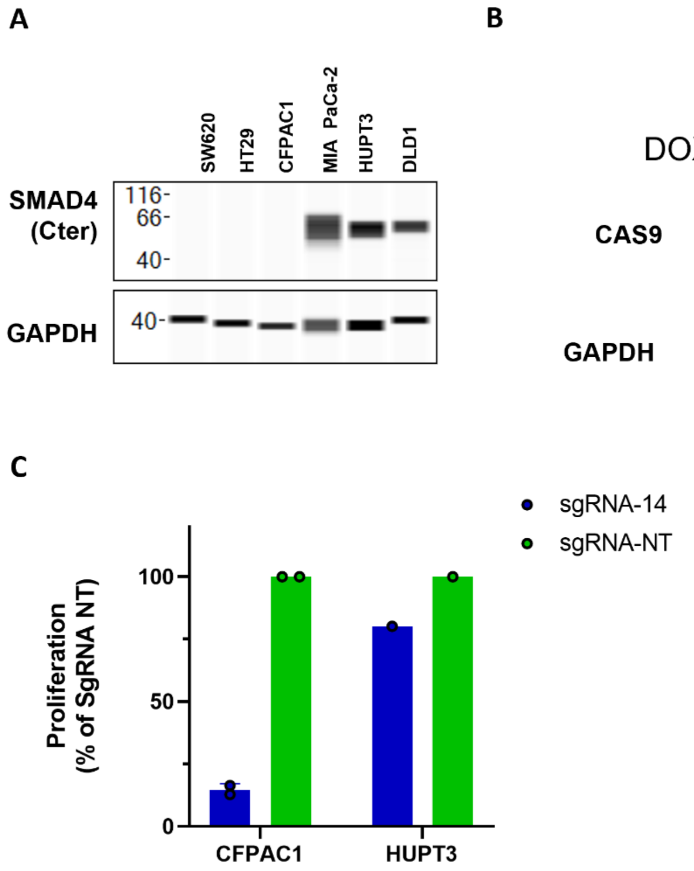
A
SW620
HT29
CFPAC1
MIA PaCa-2
HUPT3
DLD1
SMAD4 (Cter)
116-
66-
40-
GAPDH
40-
B
CFPAC1 pCM3586 C1
HUPT3 pCM3586 C33
DOX
-
+
CAS9
180-
116-
66-
GAPDH
40-
C
sgRNA-14
sgRNA-NT
Proliferation (% of SgRNA NT)
100
50
0
CFPAC1
HUPT3

Figure S6: Validation of RAB10 susceptibility in two additional cell lines. A, RAB10 expression assessed with western blot in a panel of cell lines. CFPC1 and HUPT3 cell lines are SMAD4-negative and SMAD4-positive respectively**.** B, Levels of Cas9 protein detected by the Sally Sue Simple Western system, in response to doxycycline treatment (exposure at 1ug/mL for 3 days) in the two clones CFPAC1_C1 and HUPT3_C33. **C,** The SMAD4-negative CFPAC1 and the SMAD4-positive HUPT3 cell lines were transfected with pCM3586 to express Cas9 under doxycycline treatment. Clonal isolation was performed. Based on Cas9 expression level, HUPT3 pCM3586 C33 and CFPAC1 pCM3586 C1 were chosen and infected with lentivirus expressing sgRAB10 #14. Proliferation assays were performed under doxycycline treatment as described in the article. Briefly, cells were seeded in T25 flasks at $3x10^5$ cells/flask. They were counted and split regularly under doxycycline treatment for 20 days. The total cell number reached after 20 days was estimated using the population doublings that were calculated all along the experiments at each harvest steps. The proliferation percentage with SD of each clone is plotted according to the proliferation of the counterpart control cells having the non-targeting sgRNA.
