# Supplementary material for: Genome-wide CRISPR Screen Reveals RAB10 as a Synthetic Lethal Gene in Colorectal and Pancreatic Cancers Carrying SMAD4 Loss
Source: Cancer Res Commun. 2023 May 4;3(5):780–92. doi: 10.1158/2767-9764.CRC-22-0301 (PMC10158796; doi:10.1158/2767-9764.CRC-22-0301)
Supplement: Supplementary Figure 7 — Proteins level assessment by western blotting [file crc-22-0301-s14.pdf]

Figure S7

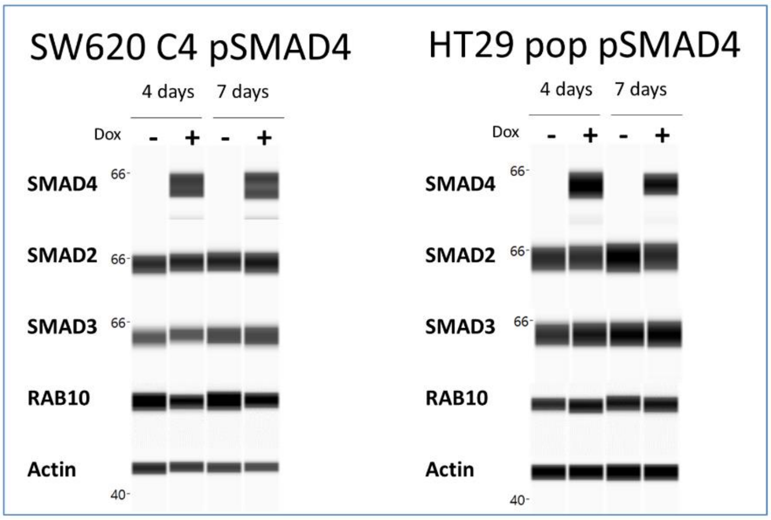
SW620 C4 pSMAD4
HT29 pop pSMAD4
4 days
7 days
Dox
- + - +
SMAD4
SMAD2
SMAD3
RAB10
Actin
66
40

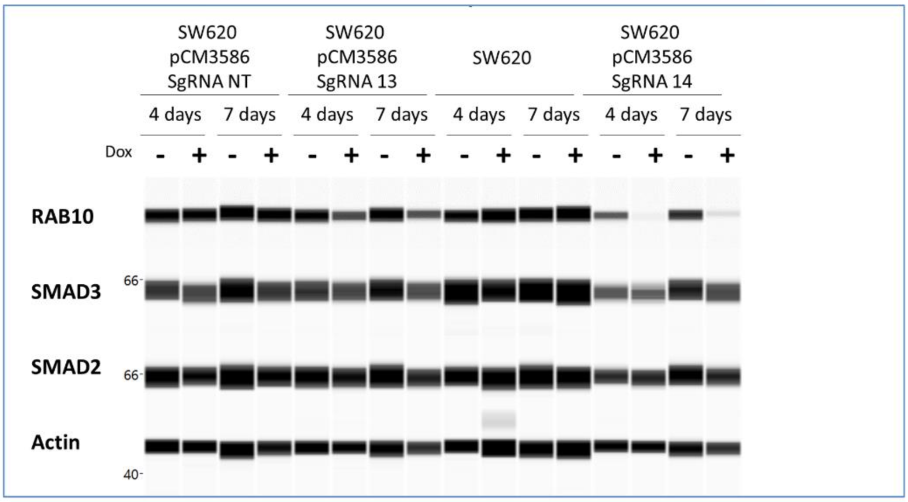
SW620 pCM3586 SgRNA NT
SW620 pCM3586 SgRNA 13
SW620
SW620 pCM3586 SgRNA 14
4 days
7 days
Dox
RAB10
SMAD3
SMAD2
Actin
66
40

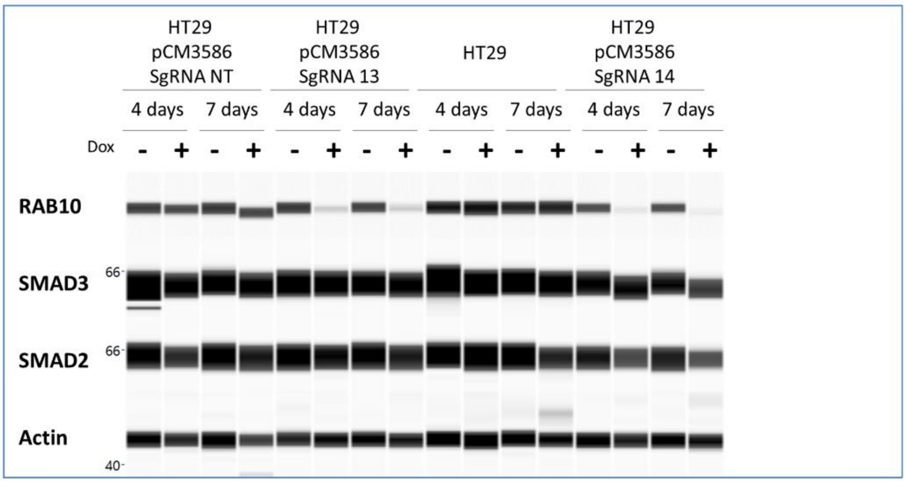
HT29 pCM3586 SgRNA NT
HT29 pCM3586 SgRNA 13
HT29
HT29 pCM3586 SgRNA 14
4 days
7 days
Dox
RAB10
SMAD3
SMAD2
Actin
66
40

Figure S7: Proteins level assessment by western blotting

To check whether SMAD4 re-introduction alters RAB10 protein levels we performed western blots on SW620 pSmad4 and HT29 pSmad4 growing under doxycycline treatment or not.To determine the impact of RAB10 KO on SMAD4, SMAD3 and SMAD2 protein levels, we also performed western blots on SW620 pCM3586 and HT29 pCM3586, to express Cas9 under doxycycline treatment, infected with sgRNA against RAB10 (sgRNA #13 and sgRNA #14) or control non-targeting sgRNA (sgRNA #NT), SW620 and HT29.
