# Supplementary material for: Genome-wide CRISPR Screen Reveals RAB10 as a Synthetic Lethal Gene in Colorectal and Pancreatic Cancers Carrying SMAD4 Loss
Source: Cancer Res Commun. 2023 May 4;3(5):780–92. doi: 10.1158/2767-9764.CRC-22-0301 (PMC10158796; doi:10.1158/2767-9764.CRC-22-0301)
Supplement: Supplementary Figure 8 — Determination of RAB10 localization [file crc-22-0301-s15.pdf]

Figure S8

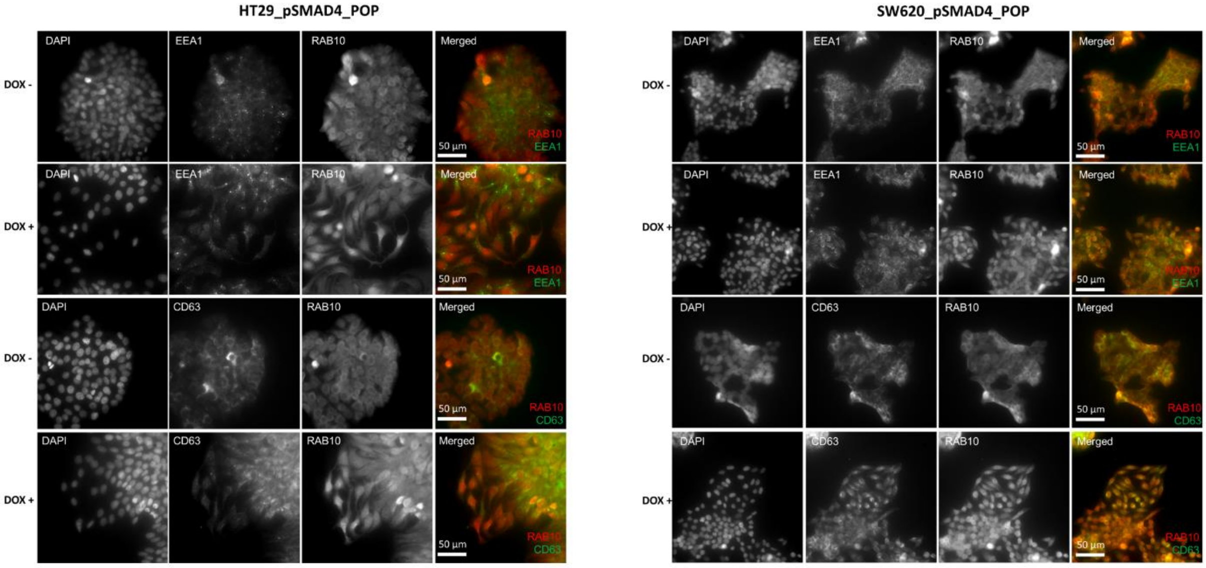
HT29_pSMAD4_POP
SW620_pSMAD4_POP
DOX -
DOX +
DAPI
EEA1
RAB10
CD63
Merged
50 µm

Figure S8: Determination of RAB10 localization

To check if Rab10 localization changes in the setting of SMAD4 loss, we performed immune fluorescence experiments on SW620 and HT29 isogenic cells in which SMAD4 is or is not present. The SMAD4-negative cell lines HT29 and SW620 with the inducible plasmid pSMAD4, were seeded at Day0, treated at Day 1 with doxycycline for 72h. Rab10 (#ab237703 1:400) was then co-stained with early (EEA1, # BD 610456 1:400) and late (CD63, # ab1318 1:400) endosome markers. Pictures were taken with a Zeiss Axio Observer fluorescent microscope.
